# Mitofusins regulate lipid metabolism to mediate the development of lung fibrosis

**DOI:** 10.1038/s41467-019-11327-1

**Published:** 2019-07-29

**Authors:** Kuei-Pin Chung, Chia-Lang Hsu, Li-Chao Fan, Ziling Huang, Divya Bhatia, Yi-Jung Chen, Shu Hisata, Soo Jung Cho, Kiichi Nakahira, Mitsuru Imamura, Mary E. Choi, Chong-Jen Yu, Suzanne M. Cloonan, Augustine M. K. Choi

**Affiliations:** 1 Division of Pulmonary and Critical Care Medicine, Joan and Sanford I. Weill Department of Medicine, Weill Cornell Medicine, New York, NY 10021 USA; 2 Department of Laboratory Medicine, National Taiwan University Hospital and National Taiwan University Cancer Center, Taipei, 10002 Taiwan; 3 Graduate Institute of Clinical Medicine, College of Medicine, National Taiwan University, Taipei, 10051 Taiwan; 4 Department of Medical Research, National Taiwan University Hospital, Taipei, 10002 Taiwan; 5 Division of Nephrology and Hypertension, Joan and Sanford I. Weill Department of Medicine, Weill Cornell Medicine, New York, NY 10021 USA; 6 Department of Internal Medicine, National Taiwan University Hospital, Taipei, 10002 Taiwan; 7 New York Presbyterian Hospital-Weill Cornell Medical Center, New York, NY 10021 USA; 8 Department of Internal Medicine, College of Medicine, National Taiwan University, Taipei, 10051 Taiwan

**Keywords:** Mitochondria, Respiratory tract diseases, Lipids

## Abstract

Accumulating evidence illustrates a fundamental role for mitochondria in lung alveolar type 2 epithelial cell (AEC2) dysfunction in the pathogenesis of idiopathic pulmonary fibrosis. However, the role of mitochondrial fusion in AEC2 function and lung fibrosis development remains unknown. Here we report that the absence of the mitochondrial fusion proteins mitofusin1 (MFN1) and mitofusin2 (MFN2) in murine AEC2 cells leads to morbidity and mortality associated with spontaneous lung fibrosis. We uncover a crucial role for MFN1 and MFN2 in the production of surfactant lipids with MFN1 and MFN2 regulating the synthesis of phospholipids and cholesterol in AEC2 cells. Loss of MFN1, MFN2 or inhibiting lipid synthesis via fatty acid synthase deficiency in AEC2 cells exacerbates bleomycin-induced lung fibrosis. We propose a tenet that mitochondrial fusion and lipid metabolism are tightly linked to regulate AEC2 cell injury and subsequent fibrotic remodeling in the lung.

## Abstract (web summary)

Mitochondria of alveolar type 2 epithelial cells (AEC2) in the lung have been suggested to play a role in the development of idiopathic pulmonary fibrosis (IPF). Here the authors show that loss of mitofusin1 and mitofusin2 in murine AEC2 cells leads to the development of lung fibrosis through the regulation of surfactant lipids.

## Introduction

Alveoli, the basic units for gaseous exchange in the lung, are composed of alveolar type 1 (AEC1) and alveolar type 2 (AEC2) epithelial cells, capillary networks, and interstitial matrix. Although AEC2 cells cover only 5% of the alveolar surface area, they are highly specialized, metabolically active cells that have a high density of sub-cellular organelles^1^. Importantly, AEC2 cells are the main progenitor cells in the alveoli, differentiating into AEC1 cells for alveolar repair, and proliferating for self-renewal^2,3^. AEC2 cells primarily function to secrete lung surfactant, a surface-active lipoprotein complex containing ~90% lipid, predominantly composed of phospholipids, particularly dipalmitoylphosphatidylcholine, pamitoyl-myristoyl-phosphatidylcholine, and phosphatidylglycerol^4,5^, along with cholesterol. Lung surfactant reduces the surface tension, preventing the alveoli from collapsing, as well as playing a critical role in immune regulation^5,6^. Lipid synthesis is essential for the production of key phospholipid components of surfactant^4,5^, and lipid metabolism relies on proper mitochondrial function^7^, along with appropriate interactions between mitochondria and endoplasmic reticulum (ER) contact sites^8^. However, little is known about the functional role of mitochondrial-dependent lipid metabolism in surfactant producing AEC2 cells.

Mitochondria form an interconnected intracellular network, altering size and shape via processes of fission and fusion which are tightly regulated to meet cellular metabolic demands^9,10^. Mitofusins, comprising mitofusin 1 (MFN1) and mitofusin 2 (MFN2), are GTPase proteins that orchestrate outer mitochondrial membrane fusion^11^. Mitochondrial fusion is required for oxidative phosphorylation, mitochondrial DNA (mtDNA) biogenesis, mitophagy regulation, and metabolic adaptation^11–14^. Global deletion of murine *Mfn1* or *Mfn2* results in embryonic lethality^11^ and alteration of murine mitofusins in specialized cells of the heart, brain and muscle leads to cardiac and neuromuscular diseases^12,13,15,16^.

Emerging evidence has demonstrated that mitochondrial damage is evident in AEC2 cells in the lungs of patients with idiopathic pulmonary fibrosis (IPF)^17,18^. IPF is a progressive and devastating lung disease with a median survival of 3–5 years associated with excessive matrix deposition in the lungs and destruction of the alveolar structure^19^. AEC2 cells from patients with IPF have enlarged and swollen mitochondria^17,18^, and higher *MFN2* mRNA expression when compared to healthy controls^20^, suggesting that mitochondrial fusion may be perturbed in these patients. However, the association between mitochondrial fission and fusion in AEC2 cells and the development of lung fibrosis remain unknown.

In this study, we evaluated the role of mitofusins in AEC2 cells. By selectively deleting MFN1 and MFN2 in murine AEC2 cells, we reveal that AEC2 cells require mitofusins for their specialized function of surfactant lipid regulation. Using high throughput targeted lipidomic analyses in combination with transcriptomic profiling, we demonstrate that MFN1 and MFN2 are crucial for regulating lipid metabolism in response to mitochondrial damage in AEC2 cells. Importantly, deletion of *Mfn1* or *Mfn2* in murine AEC2 cells promotes experimental lung fibrosis and simultaneous deletion of *Mfn1/2* in AEC2 cells not only impairs basal surfactant phospholipid and cholesterol metabolism but also leads to the development of spontaneous lung fibrosis. Transcriptomic profiling with functional enrichment analyses suggests that the impaired surfactant lipid production in *Mfn1*- or *Mfn2*-deficient AEC2 cells may be related to imbalanced regulation of purine and lipid metabolism, both of which share common upstream substrates, including glycolytic derivatives. Collectively, we provide a pathogenic mechanism linking mitochondrial damage, impaired surfactant lipid synthesis in AEC2 cells and the development of lung fibrosis.

## Results

### Altered mitochondrial shape in AEC2 cells after bleomycin

**Fig. 1:**
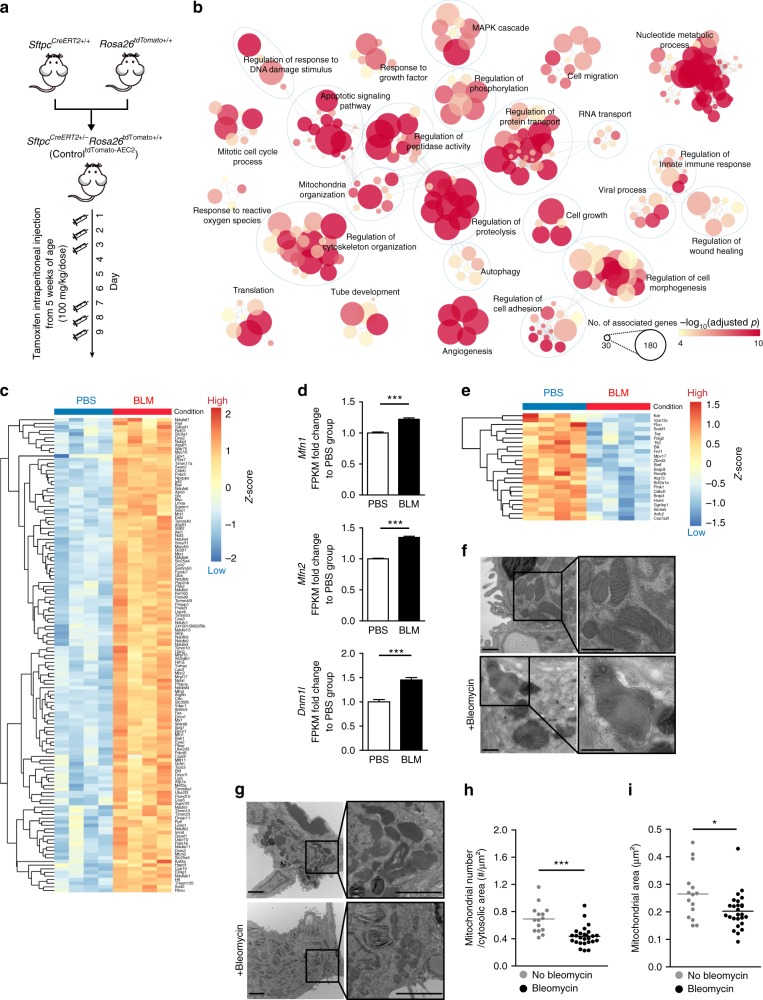
Altered mitochondrial dynamics in murine AEC2 cells in bleomycin-induced lung fibrosis. **a** Schema demonstrating the generation of mice with tamoxifen-inducible tdTomato labeling in AEC2 cells; tdTomato reporter mice (*Rosa26*^tdTomato+/+^) were crossed with *Sftpc*^CreERT2+/+^ mice. **b** A functional enrichment map generated using genes differently expressed between AEC2 cells with and those without bleomycin treatment, using the threshold of an adjusted *p* < 0.001 and a fold change >1.2. **c** Heatmap showing upregulated genes under the annotation mitochondrial organization (GO:0007005) in AEC2 cells treated with bleomycin (BLM), compared to those treated with PBS. **d** Expression of *Mfn1*, *Mfn2* and *Dnm1l* mRNA in AEC2 cells 5 days after PBS (*n* = 4 mice) or BLM (*n* = 4 mice) treatment. For each gene, the fold change of FPKM is calculated relative to the PBS group. The data are presented as mean±s.e.m. (NS, not significant; ***adjusted *p* < 0.001 vs. PBS group). **e** A heatmap showing downregulated genes under the annotation mitochondrial organization (GO:0007005) in AEC2 cells treated with bleomycin, compared to those treated with PBS. **f** Representative TEM images (50,000X) showing mitochondrial damage in AEC2 cells from *Sftpc*^CreERT2+/−^ mice before and after bleomycin treatment (scale bar 500 nm). **g**-**i** Representative TEM images (12,000X; scale bar 2 μm) (**g**) and corresponding quantification of mitochondrial number per μm^2^ of cytosolic area (**h**) and mitochondrial area (μm^2^) (**i**) in each AEC2 cell before and after bleomycin treatment. Each dot represents one AEC2 cell and the line indicates mean (before bleomycin, *n* = 15 AEC2 cells from 3 mice; after bleomycin, *n* = 26 AEC2 cells from 2 mice; *p < 0.05, ***p < 0.001, vs. no bleomycin treatment by unpaired Student’s *t* test). Source data (**c**, **d**) are provided as a Source Data file

Little is known about the role of mitochondrial dynamics in AEC2 cells or in the pathogenesis of IPF. Intra-tracheal administration of bleomycin, which induces nuclear and mitochondrial DNA strand breaks and mitochondrial respiratory chain dysfunction^21^, reproducibly induces lung fibrosis in mice and is widely used to explore the mechanisms of lung fibrosis. Transcriptomic studies in AEC2 cells from IPF lungs revealed altered genes related to mitochondrial regulation, including *MFN2* upregulation^20^. In this study, we investigated whether in vivo bleomycin administration induces similar transcriptomic responses in murine AEC2 cells. To isolate AEC2 cell populations, we generated a unique AEC2 cell reporter mouse by crossing *Sftpc*^*CreERT2*+/+^ mice with *ROSA26*^tdTomato+/+^ mice, creating mice with tamoxifen-inducible tdTomato florescence in AEC2 cells (*Sftpc*^CreERT2+/−^*ROSA26*^tdTomato+/−^, referred to as control^tdTomato-AEC2^) (Fig. 1a)^22^. Five days after bleomycin treatment AEC2 cells were isolated for RNA next-generation sequencing (RNA-seq) utilizing flow cytometric cell sorting of tdTomato positive cells (Supplementary Fig. 1a). Functional enrichment analyses of differential transcripts between AEC2 cells isolated from mice treated with bleomycin and AEC2 cells from controls identified several significantly altered mitochondrial and metabolic cellular pathways, including mitochondrial organization, apoptotic signaling, nucleotide metabolic process, regulation of protein transport, RNA transport, and autophagy (Fig. 1b and Supplementary Fig. 2e). Examination of genes included in the mitochondrial organization annotation revealed the upregulation of genes involved in mitochondrial dynamic regulation (such as *Mfn1*, *Mfn2*, *Dnm1l*, and *March5*) mitochondrial apoptotic control (such as *Bcl2l1*, *Mcl1*, *Bax*, *Bid*, and *Bak1*), and mitochondrial oxidative phosphorylation (such as *Ndufb6*, *Ndufs6* and *Ndufa12* for complex I, *Sdhd* for complex II, *Cyc1*, *Cycs*, *Uqcrb*, and *Uqcrq* for complex III, and *Cox5a*, *Cox5b*, *Cox6a1* and *Cox7c* for complex IV) (Fig. 1c, d and Supplementary Data 1), while there was downregulation of genes involved in mitophagy (*Pink1*, *Bnip3*, and *Atg13*) (Fig. 1e). This data highlighted similar transcriptomic responses between murine AEC2 cells after bleomycin treatment and human AEC2 cells from IPF lungs^17,20^.

We next examined mitochondrial ultrastructural changes in AEC2 cells in the murine model of bleomycin-induced lung fibrosis through transmission electron microscopy (TEM). AEC2 cells of mice exposed to bleomycin (8 days post treatment) showed swollen mitochondria with disrupted cristae (Fig. 1f and Supplementary Fig. 2a), which, when compared to the controls, had significantly decreased mitochondrial number (Fig. 1h) and area (Fig. 1g, i and Supplementary Fig. 2b). Immunoblotting showed decreased OPA1 (for optic atrophy 1) protein levels with no change in DRP1 (for dynamin-1-like protein), MFN1 or MFN2 expression in AEC2 cells 8 days post bleomycin exposure (Supplementary Fig. 2c, d). Collectively, the data suggested that bleomycin alters mitochondrial dynamics, leading to mitochondrial fragmentation, and also alters the expression of several genes related to mitochondrial regulation in AEC2 cells.

### Loss of *Mfn1* or *Mfn2* in AEC2 cells promotes lung fibrosis

**Fig. 2:**
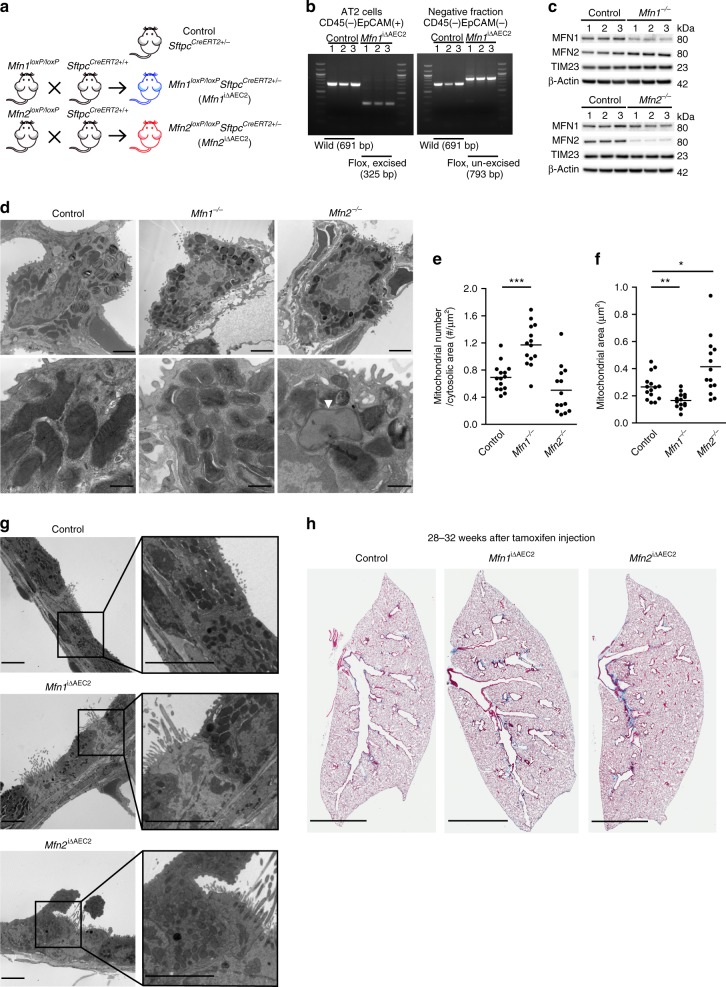
Mice with AEC2 cell-specific deletion of *Mfn1* or *Mfn2*. **a** Schema demonstrating the generation of AEC2 cell-specific mice deficient in *Mfn1* or *Mfn2* using a tamoxifen-inducible *Sftpc*-promoter driven CreERT2. *Sftpc*^CreERT2+/−^ mice were used as controls. **b** Genotyping of DNA extracted from CD45(−)EpCAM(+) cells and CD45(-)EpCAM(−) cells isolated from control and *Mfn1*^iΔAEC2^ mice 6 weeks after tamoxifen injection (*n* = 3 mice per group). **c** Representative immunoblots of AEC2 cell lysates obtained 3 weeks after tamoxifen-induced deletion, showing decreased protein levels of MFN1 or MFN2 in the respective knockout cells (*n* = 3 mice per group). **d** Representative TEM images (upper row, ×12,000, scale bar 2 μm; lower row, ×50,000, scale bar 500 nm) show mitochondrial ultrastructural changes in *Mfn1*^−/−^ and *Mfn2*^−/−^ AEC2 cells (*n* = 3 mice per group) with disrupted cristae marked with white arrowheads. **e**, **f** Quantification of (**d**); mitochondrial number per μm^2^ of cytosolic area (**e**) and mitochondrial area (μm^2^) (**f**) in each AEC2 cell, using TEM images (×12,000). Each dot represents one AEC2 cell, and the line indicates mean (control AEC2 cells *n* = 15, *Mfn1*^−/−^ AEC2 cells *n* = 14; *Mfn2*^*−/−*^ AEC2 cells *n* = 14, from a total of 3 mice per group; *p < 0.05, **p < 0.01, ***p < 0.001, vs. control by unpaired Student’s *t* test). **g** Representative TEM images (×5000) of the bronchial epithelium in control (*Sftpc*^CreERT2+/−^), *Mfn1*^iΔAEC2^ and *Mfn2*^iΔAEC2^ mice (*n* = 3 mice per group; scale bar 5 μm). **h** Representative Masson’s trichrome-stained sections (×100 magnification) of murine left lung at 28–32 weeks post tamoxifen-induced deletion (control mice, *n* = 20; *Mfn1*^iΔAEC2^ mice, *n* = 10; *Mfn2*^iΔAEC2^ mice, *n* = 9; scale bar 3 mm). Source data (**c**, **e**, **f**) are provided as a Source Data file

To elucidate the precise function of MFN1 and MFN2 in AEC2 cells, we conditionally deleted *Mfn1* and *Mfn2* genes in murine AEC2 cells. Specifically, genetically modified mice harboring *Mfn1* or *Mfn2* flanked by two *loxP* sites were crossed with *Sftpc*^*CreERT2+/+*^ mice (Fig. 2a)^12^. AEC2 cells were isolated from murine lungs, through CD45-negative selection and subsequent EpCAM-positive selection (Supplementary Fig. 1b-e)^23^. Tamoxifen treatment resulted in the selective deletion of *Mfn1* and *Mfn2* genes in AEC2 cells (*Mfn1*^loxP/loxP^*Sftpc*^CreERT2+/−^ (*Mfn1*^iΔAEC2^) and *Mfn2*^loxP/loxP^*Sftpc*^CreERT2+/−^ (*Mfn2*^iΔAEC2^) mice respectively)^24^, as confirmed by genotyping (Fig. 2b) and immunoblotting (Fig. 2c). To access for potential off-target toxicity by CreERT2, heterozygous *Sftpc*^CreERT2+/−^ transgenic mice were used as controls.

Given the pivotal role for MFN1 and MFN2 in regulating mitochondrial fusion, we first evaluated mitochondrial ultrastructural changes in *Mfn1*^*−/−*^ and *Mfn2*^*−/−*^ AEC2 cells. TEM analysis confirmed that at baseline *Mfn1*^*−/−*^ AEC2 cells displayed fragmented mitochondria with decreased mitochondrial area, increased mitochondrial number, but normal cristae (Fig. 2d-f and Supplementary Fig. 3b). In contrast, abnormally enlarged mitochondria with irregular and disrupted cristae were seen in the *Mfn2*^*−/−*^ AEC2 cells (Supplementary Fig. 3a). These mitochondrial morphological changes were restricted to AEC2 cells and were not observed in other lung cells, such as bronchial epithelial cells (Fig. 2g). To confirm the above findings, we depleted MFN1 or MFN2 in the murine AEC2 cell line MLE 12 through shRNA lentiviral transduction (Supplementary Fig. 4a). Loss of MFN1 induced more mitochondrial fragmentation than loss of MFN2 in MLE 12 cells (Supplementary Fig. 4b). Depletion of MFN1 or MFN2 in the human AEC2 cell line A549 also altered mitochondrial morphology (Supplementary Fig. 4c, d). Ultrastructural examination further revealed the increased presence of abnormal mitochondria (swollen, irregular cristae) in MFN2-deficient MLE 12 cells (Supplementary Fig. 4e). We surmised that such morphological changes might be indicative of a failure of MFN2-deficient MLE 12 cells to activate mitophagy for mitochondrial quality control^14^. To verify the importance of MFN2 in mitophagy regulation, we generated a mitophagy reporter system using mtKeima fluorescent protein^25^. We found mitophagy induced by oligomycin and antimycin A^25^ was only mildly suppressed by MFN1 deficiency, but markedly suppressed by MFN2 deficiency in MLE 12 cells (Supplementary Fig. 4f). Collectively, these data show that loss of either MFN1 or MFN2 alters mitochondrial morphology and turnover in AEC2 cells.

**Fig. 3:**
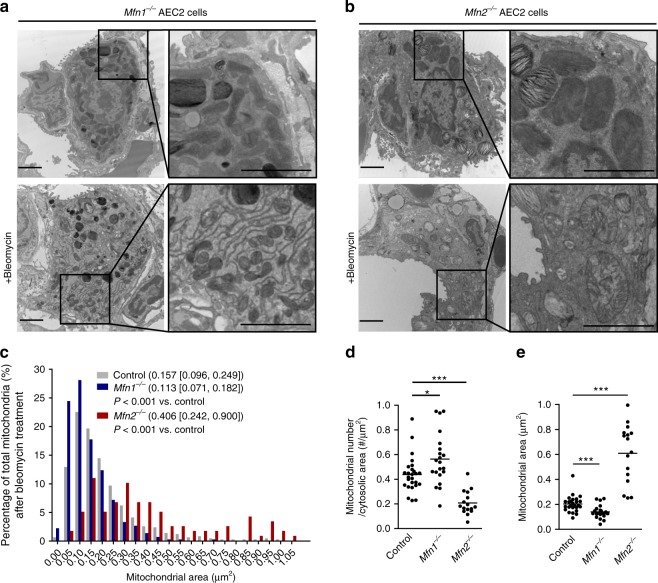
Mitofusin-deficient AEC2 cells susceptible to bleomycin-induced mitochondrial damage. **a**, **b** Representative TEM images (12,000X; scale bar 2 μm) in *Mfn1*^−/−^ (**a**) or *Mfn2*^−/−^ (**b**) AEC2 cells before (*n* = 3 mice) and after bleomycin treatment (*n* = 2 mice). **c**–**e** Quantification of mitochondria area of each mitochondrion (control *n* = 521 mitochondria, *Mfn1*^−/−^ *n* = 464 mitochondria, *Mfn2*^−/−^ *n* = 119 mitochondria; data presented as the median [interquartile range], and the comparison performed by Mann–Whitney *U* test) (**c**), and mitochondrial number per μm^2^ of cytosolic area (**d**) and mitochondrial area (μm^2^) (**e**) in each AEC2 cell (**d**, **e**, each dot represents one AEC2 cell; *p < 0.05, ***p < 0.001, vs. control by unpaired Student’s *t* test), using TEM images (12,000X) of control, *Mfn1*^−/−^ or *Mfn2*^−/−^ AEC2 cells 8 days after bleomycin treatment (control AEC2 cells *n* = 26, *Mfn1*^−/−^ AEC2 cells *n* = 21, *Mfn2*^−/−^ AEC2 cells *n* = 16, from 2 mice per group). Source data (**c**–**e**) are provided as a Source Data file

Despite evidence of mitochondrial dysfunction, *Mfn1*^iΔAEC2^ and *Mfn2*^iΔAEC2^ mice continued to thrive at 28–32 weeks post tamoxifen treatment, without remarkable lung pathologies (Fig. 2h). To investigate whether deficiency of MFN1 or MFN2 in AEC2 cells altered the development of lung fibrosis after bleomycin treatment, *Mfn1*^iΔAEC2^ and *Mfn2*^iΔAEC2^ mice were instilled with bleomycin. TEM analysis showed that *Mfn1* or *Mfn2* deletion enhanced bleomycin-induced mitochondrial damage in AEC2 cells (Fig. 3a, b). After bleomycin treatment, compared to control AEC2 cells, *Mfn1*^−/−^ AEC2 cells showed decreased mitochondrial area and increased mitochondrial number, while *Mfn2*^−/−^ AEC2 cells showed increased mitochondrial area and decreased mitochondrial number (Fig. 3c–e and Supplementary Fig. 5a–f**)**. The data suggested that *Mfn1* deletion leads to excessive mitochondrial fragmentation, while *Mfn2* deletion leads to swollen mitochondria in AEC2 cells after bleomycin treatment. We also found that bleomycin treatment and deletion of *Mfn1* or *Mfn2* did not alter the amount of mtDNA present in AEC2 cells (Supplementary Fig. 5g)^13^.

**Fig. 4:**
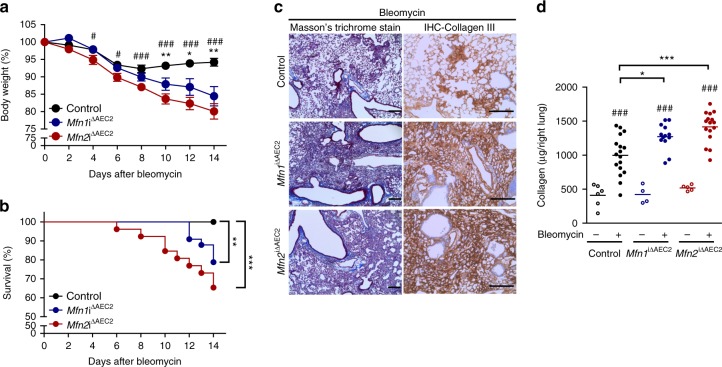
MFN1 or MFN2 deficiency in AEC2 cells promotes bleomycin-induced lung fibrosis. **a** Body weight changes of control (*n* = 18), *Mfn1*^iΔAEC2^ (*n* = 13) and *Mfn2*^iΔAEC2^ (*n* = 16) mice after bleomycin treatment. Data are mean±s.e.m. (results from 3 independent experiments; *Mfn1^iΔAEC2^ vs. control, ^#^*Mfn2*^iΔAEC2^ vs. control; * and ^#^, *p* < 0.05, ** and ^##^, *p* < 0.01, ^###^*p* < 0.001, by unpaired Student’s *t* test). **b** Kaplan–Meier survival curves of control (*n* = 32), *Mfn1*^iΔAEC2^ (*n* = 33) and *Mfn2*^iΔAEC2^ (*n* = 26) mice after bleomycin treatment (results from 3 independent experiments; **p < 0.01, ***p < 0.001, by log-rank test). **c** Masson’s trichrome staining (left panel, ×100 magnification, scale bar 200 μm) and IHC staining of collagen III (right panel, ×200 magnification, scale bar 200 μm) in lung sections of control, *Mfn1*^iΔAEC2^ and *Mfn2*^iΔAEC2^ mice14 days after bleomycin treatment (control mice *n* = 10, *Mfn1*^iΔAEC2^ mice *n* = 12, *Mfn2*^iΔAEC2^ mice *n* = 3). **d** Acid-soluble collagen levels in the right lung from control (PBS *n* = 6, bleomycin *n* = 17), *Mfn1*^iΔAEC2^ (PBS *n* = 4, bleomycin *n* = 13) and *Mfn2*^iΔAEC2^ (PBS *n* = 5, bleomycin *n* = 16) mice 14 days after PBS or bleomycin treatment, quantified by Sircol assay. The line indicates mean (^#^, bleomycin vs. PBS, * vs. control mice; *p < 0.05, *** and ^###^*p* < 0.001 by one-way ANOVA with post-hoc Bonferroni test). Source data (**a**, **b**, **d**) are provided as a Source Data file

In the bleomycin model, weight loss occurs with disease progression and correlates with the severity of lung fibrosis^26^. We found, compared to control mice, both *Mfn1*^iΔAEC2^ and *Mfn2*^iΔAEC2^ mice demonstrated persistent weight loss (Fig. 4a) and increased mortality (Fig. 4b) after bleomycin exposure. Lungs from *Mfn1*^iΔAE2^ and *Mfn2*^iΔAE2^ mice also showed more intense Masson’s trichrome staining of fibrotic regions, along with increased immunohistochemical (IHC) staining for collagen III (Fig. 4c). Quantification of acid-soluble collagen showed that *Mfn1*^iΔAEC2^ and *Mfn2*^iΔAEC2^ mice, compared to the control, had more lung collagen deposition after bleomycin treatment (Fig. 4d). Compared to the control, *Mfn1*^iΔAEC2^ or *Mfn2*^iΔAEC2^ mice had similar protein levels in bronchoalveolar lavage fluid (BALF), and did not have increased inflammatory cell infiltrates after bleomycin treatment (Supplementary Fig. 6a–c).

### Loss of AEC2 cell *Mfn1/2* induces spontaneous lung fibrosis

**Fig. 5:**
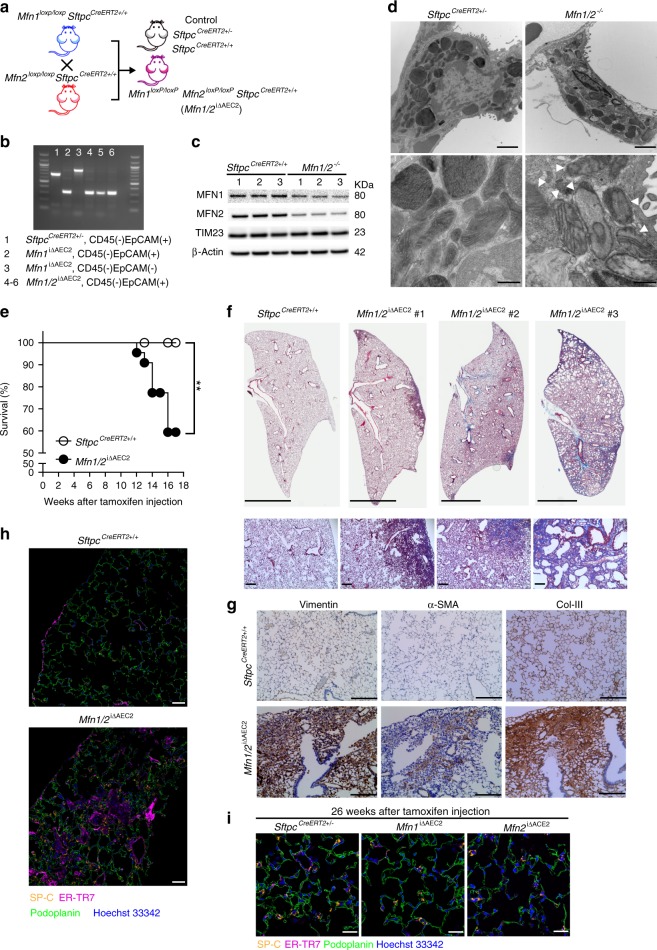
*Mfn1/2*^iΔAEC2^ mice develop spontaneous lung fibrosis. **a** Schema demonstrating the generation of mice with AEC2 cell-specific tamoxifen-inducible deletion of *Mfn1/2*. *Sftpc*^*CreERT2+/+*^ or *Sftpc*^*CreERT2*^^+/−^ mice were used as controls. **b** Genotyping of CD45(-)EpCAM(+) cells isolated from *Mfn1/2*^iΔAEC2^ mice (*n* = 3 mice; lane 1 to lane 3 serves as the positive control). **c** Representative immunoblots of AEC2 cell lysates obtained 6 weeks after tamoxifen-induced deletion, showing decreased protein levels of both MFN1 and MFN2 in the *Mfn1/2*^−/−^ AEC2 cells (*n* = 3 mice per group). **d** Representative TEM images (upper row, ×12,000; lower row, ×50,000) show mitochondrial ultrastructural changes in *Sftpc*^*CreERT2*^^+/−^ and *Mfn1/2*^−/−^ AEC2 cells (*n* = 3 mice per group) with disrupted cristae marked with white arrowheads (scale bar, upper row 2 μm, lower row 500 nm. **e** Kaplan–Meier survival curves of *Mfn1/2*^iΔAEC2^ (*n* = 22) and *Sftpc*^*CreERT2+/+*^ (*n* = 23) mice (*p* < 0.01 by log-rank test). **f** Representative Masson’s trichrome-stained lung sections (upper panel, ×100 magnification; lower panel, ×200 magnification) 17 weeks post tamoxifen-induced deletion (*Sftpc*^*CreERT2+/+*^ mice *n* = 6; *Mfn1/2*^iΔAEC2^ mice *n* = 11; scale bar, upper panel 4 mm, lower panel 200 μm). **g** Representative IHC staining of vimentin, alpha-smooth muscle actin (α-SMA), and collagen III (Col-III) (×200 magnification; *n* = 3 mice per group; scale bar 200 μm). **h** Representative immunofluorescent staining of 5x5 tiled confocal images (using ×40 objective) of frozen murine lung sections stained for podoplanin (green), surfactant protein-C (SP-C) (yellow), ER-TR7 (magenta), and Hoechst 33342 stain (blue) (*n* = 3 mice per group; scale bar 50 μm). **i** Representative immunofluorescence staining confocal images of podoplanin (green), SP-C (yellow), ER-TR7 (magenta) and Hoechst 33342 nuclear stain (blue) using lung sections of *Sftpc*^*CreERT2*^^+/−^, *Mfn1*^iΔAEC2^ and *Mfn2*^iΔAEC2^ mice (*n* = 2 mice per group; scale bar 20 μm). Source data (**c**, **e**) are provided as a Source Data file

Single-gene deletion of *Mfn1* or *Mfn2* in AEC2 cells exacerbated bleomycin-induced lung fibrosis, but at baseline did not cause any obvious lung pathology. We hypothesized that MFN1 and MFN2 may compensate for the loss of each other to maintain the basal function of AEC2 cells. We therefore generated mice in which both *Mfn1* and *Mfn2* were simultaneously deleted in AEC2 cells (*Mfn1*^*loxP/loxP*^*Mfn2*^*loxP/loxP*^*Sftpc*^*CreERT2+/+*^ (*Mfn1/2*^iΔAEC2^)) and confirmed by genotyping and immunoblotting (Fig. 5a-c). TEM analysis of mitochondrial ultrastructure showed loss of both *Mfn1/2* led to increased mitochondrial area (Supplementary Fig. 7a-c), decreased mtDNA copy number (Supplementary Fig. 7d)^13,16^, and considerable accumulation of abnormal mitochondria with disrupted cristae in AEC2 cells (Fig. 5d and Supplementary Fig. 7e). Strikingly, 36.4% of mice deficient in both *Mfn1* and *Mfn2* in AEC2 cells died by 16 weeks post tamoxifen treatment, with equal penetrance in both sexes (Fig. 5e). Morphological and pathological assessment of lung sections from surviving (~17 weeks post tamoxifen treatment) *Mfn1/2*^iΔAEC2^ mice revealed significant increases in Masson trichrome positive staining for collagen deposition, indicative of lung fibrosis (Fig. 5f). All the remaining surviving mice which displayed signs of respiratory distress (i.e. gasping) developed severe and widespread fibrosis involving both lungs. Such trichrome positive regions principally extended from the sub-pleural parenchyma, with no predilection toward right or left lungs, and the pattern of progression resembled those observed in human IPF^19^. IHC staining of the fibrotic zone showed strong positivity for several fibrotic markers, including vimentin, α-smooth muscle actin, and collagen III (Fig. 5g). Immunofluorescent staining of *Mfn1/2*^iΔAEC2^ murine lungs also demonstrated increased localization of ER-TR7 positive fibroblastic aggregates (Fig. 5h), which were surrounded by AEC2 cells (Supplementary Fig. 8), possibly indicative of more fibrosis^27^. Morphological features of fibrosis or distinct fibroblastic aggregations were not observed in the lungs of *Mfn1*^iΔAEC2^, *Mfn2*^iΔAEC2^, *Sftpc*^*CreERT2*^^+/−^ or *Sftpc*^*CreERT2+/+*^ mice (Fig. 5i). Collectively, the above results show that *Mfn1/2*^iΔAEC2^ mice develop spontaneous lung fibrosis, which is associated with extensive mitochondrial damage in AEC2 cells.

### MFN1/2 regulate lipid metabolism in AEC2 cells

Deletion of either *Mfn1* or *Mfn2* in murine AEC2 cells aggravated bleomycin-induced lung fibrosis, while deletion of both induced spontaneous lung fibrosis. Given that both bleomycin and depletion of MFN1/2 can impair mitochondrial respiration through mtDNA damage^13,21^, we next examined whether mtDNA damage-associated mitochondrial bioenergetic failure^28^ in AEC2 cells can directly induce lung fibrosis. Mice with a mutation in mtDNA polymerase γ (*PolgA*^D257A/D257A^), the polymerase responsible for proofreading during mtDNA replication, display accumulation of mtDNA mutations and failure of mitochondrial bioenergetics, leading to premature aging and shortened lifespan^28,29^. We found that *PolgA*^D257A/D257A^ mice had increased swollen mitochondria in AEC2 cells (Supplementary Fig. 9), but did not demonstrate any pathological changes indicative of the development of spontaneous lung fibrosis, up to the age of 36–40 weeks. These findings indicate that the phenotype of lung fibrosis may be specific to *Mfn1/2*^iΔAEC2^ mice and independent to bioenergetic failure alone.

To assess AEC2 cell injury and proliferation in the bleomycin-induced lung fibrosis mouse model and to explore the biological processes affected in AEC2 cells after deletion of *Mfn1* or *Mfn2*, we generated *Mfn1*^*loxP/loxP*^*Sftpc*^*CreERT2*^^+/−^*ROSA26*^*tdTomato*^^+/−^ (*Mfn1*^iΔAEC2/tdTomato-AEC2^) and *Mfn2*^*loxP/loxP*^*Sftpc*^*CreERT2*^^+/−^*ROSA26*^*tdTomato*^^+/−^ (*Mfn2*^iΔAEC2/tdTomato-AEC2^) mice (Supplementary Fig. 10a), and performed transcriptomic profiling in *Mfn1-* and *Mfn2*-deficient AEC2 cells. The generation of these mice allowed for tamoxifen-inducible tdTomato fluorescent labeling in *Mfn1-* and *Mfn2*-deficient AEC2 cells^24^, and was confirmed by demonstrating excision of the floxed allele after tamoxifen injection (Supplementary Fig. 10b, c). In the bleomycin-induced lung fibrosis model, we did not observe that *Mfn1*^−/−^ or *Mfn2*^−/−^ AEC2 cells had significantly increased cell death 5 days after bleomycin administration (Supplementary Fig. 11a–d). The expression levels of the proliferative marker *Mki67* (RNA-Seq data) in AEC2 cells did not significantly increase (Supplementary Fig. 11e), and was not significantly different between controls and *Mfn1*^iΔAEC2^ and *Mfn2*^iΔAEC2^ mice 5 days after bleomycin exposure (Supplementary Data 3). We further observed minimal Ki67 positive nuclear staining in tdTomato-positive AEC2 cells, 10 days after bleomycin treatment (Supplementary Fig. 11f).

Transcriptomic profiling at baseline showed *Mfn2* deletion, compared to *Mfn1* deletion, resulted in more robust changes in gene expressions in AEC2 cells (Supplementary Fig. 10d and Supplementary Data 2). Specifically, *Mfn2*^−/−^ AEC2 cells, but not *Mfn1*^−/−^ AEC2 cells, activated genes involved in ATF5-mediated mitochondrial unfolded protein responses (UPR^MT^) (*Atf5*, *Lonp1*, *Clpp*, and *Hspa9*), ATF4-mediated stress pathways (*Atf4*, *Ddit3*, *Asns*, *Chac1*, *Pck2*, and *Trib3*), along with genes involved in de novo serine/glycine synthesis pathways (*Phgdh*, *Psat1*, *Shmt2*) (Supplementary Fig. 10e)^30,31^. Neither *Mfn1*^−/−^ or *Mfn2*^−/−^ AEC2 cells activated genes related to UPR^ER^, such as *Hspa5*, *Atf6*, *Pdia2*, *Ero1l*, *Xbp1*, *Hsp90b1*, and *Calr*. Furthermore, in addition to organelle fusion, the common metabolic biological processes revealed by functional enrichment analyses included lipid localization, nucleotide phosphate metabolic process, and alcohol metabolic process (Supplementary Fig. 10f).

**Fig. 6:**
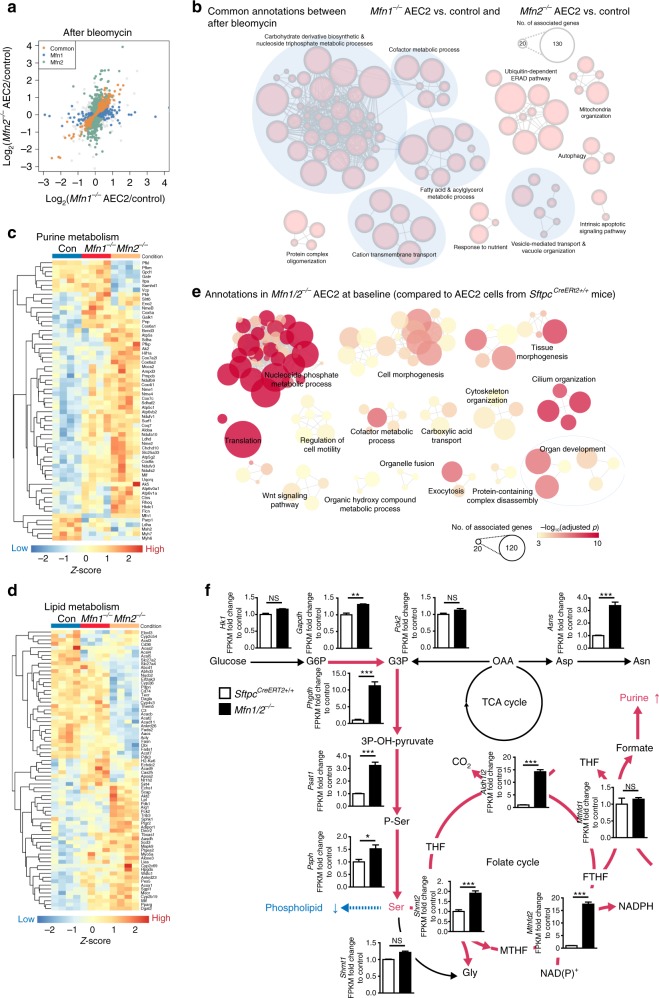
Purine metabolism is upregulated in *Mfn1*^−/−^ and *Mfn2*^−/−^ AEC2 cells after bleomycin treatment. **a** Scatterplot showing genes (orange) that are differentially expressed (adjusted *p* < 0.05) and have the same regulation direction in both *Mfn1*^−/−^ and *Mfn2*^−/−^ AEC2 cells after bleomycin treatment, compared to the control. **b** A functional enrichment map to show the common GO terms enriched on differentially expressed genes of *Mfn1*^−/−^ and *Mfn2*^−/−^ AEC2 cells after bleomycin treatment. **c**, **d** Heatmaps to show genes related to purine metabolism under the annotation purine ribonucleoside triphosphate metabolic process (GO: GO:0009205) (**c**) and genes related to lipid metabolism under the annotation fatty acid metabolic process (GO:0006631) (**d**), based on the functional enrichment results (**b**). **e** A functional enrichment map generated using genes differently expressed between *Mfn1/2*^−/−^ AEC2 cells and control AEC2 cells, using the threshold of an adjusted *p* < 0.05. **f** Differentially regulated genes related to glycolysis, asparagine (Asn) synthesis, de novo serine/glycine synthesis, and mitochondrial one-carbon metabolism in *Sftpc*^*CreERT2+/+*^ versus *MFN1/2*^−/−^ AEC2 cells. For each gene, the fold change of FPKM is calculated relative to *Sftpc*^*CreERT2+/+*^ control (G6P glucose-6-phosphate, G3P glyceraldehyde-3-phosphate, OAA oxaloacetate, Asp aspartate, Asn asparagine, 3P-OH-pyruvate 3-phosphohydropyruvate, P-ser 3-phosphoserine, Ser serine, Gly glycine, THF tetrahydrofolate, MTHF methyltetrahydrofolate, FTHF formyltetrahydropholate). The data are presented as mean±s.e.m. (NS not-significant; *adjusted *p* < 0.05, **adjusted *p* < 0.01, ***adjusted *p* < 0.001 vs. *Sftpc*^*CreERT2+/+*^)

In the bleomycin-induced lung fibrosis model, we found increased common genes which were regulated in both *Mfn1*^−/−^ and *Mfn2*^−/−^ AEC2 cells (Fig. 6a and Supplementary Data 3). Functional enrichment analyses of these common genes identified fatty acid and acylglycerol metabolic process, carbohydrate derivative biosynthetic and nucleoside triphosphate metabolic process and cofactor metabolic process as the major metabolic processes affected in both *Mfn1*^−/−^ and *Mfn2*^−/−^ AEC2 cells after bleomycin treatment (Fig. 6b). Examination of genes included in the carbohydrate derivative biosynthetic and nucleoside triphosphate metabolic process, we found upregulation of genes involving oxidative respiratory complexes, and genes involving purine metabolism, particularly nucleoside diphosphate kinase, adenylate kinase, polyribonucleotide nucleotidyltransferase, and adenosine monophosphate deaminase (Fig. 6c). Moreover, a number of genes involved in lipid metabolism were differentially regulated between control and *Mfn1*^−/−^ or *Mfn2*^−/−^ AEC2 cells (Fig. 6d), with the downregulation of genes related to fatty acid synthesis, long-chain fatty acid transport, fatty acid activation, elongation and modification (Supplementary Fig. 11g).

The above transcriptomic results suggest upregulated purine metabolism and downregulated lipid metabolism in *Mfn1*^−/−^ or *Mfn2*^−/−^ AEC2 cells after bleomycin. Considering *Mfn1/2*^iΔAEC2^ mice develop spontaneous lung fibrosis, we evaluated whether the transcriptomic response in *Mfn1/2*^−/−^ AEC2 cells at baseline resembled those in the *Mfn1*^−/−^ or *Mfn2*^−/−^ AEC2 cells after bleomycin treatment, and whether *Mfn1/2*^−/−^ AEC2 cells had functional annotations in common with control AEC2 cells after bleomycin treatment. Functional enrichment analyses comparing isolated AEC2 cell transcripts from *Mfn1/2*^iΔAEC2^ and *Sftpc*^*CreERt2+/+*^ (control) mice showed that *Mfn1/2*-deletion markedly affected nucleoside phosphate metabolic process in AEC2 cells (Fig. 6e and Supplementary Data 4), of which purine metabolism is the major biological pathway included in this annotation. Compared to control AEC2 cells, *Mfn1/2*-deletion in AEC2 cells activated profound ATF5-mediated UPR^MT^, and ATF4-mediated stress response pathways (Supplementary Fig. 12a), along with activation of de novo glycolytic serine/glycine synthesis pathways and mitochondrial one-carbon metabolism (Fig. 6f)^30,31^, Together, the results from transcriptomic analyses suggested upregulation of purine metabolism in *Mfn1/2*^−/−^ AEC2 cells.

We next compared the overlapping genes identified in *Mfn1/2*^−/−^ AEC2 cells with those identified AEC2 cells after bleomycin treatment (Supplementary Fig. 12b). Functional enrichment analysis of these overlapping genes confirmed that altered purine metabolism and oxidative phosphorylation are common biological processes altered in *Mfn1/2*^−/−^ AEC2 cells and in AEC2 cells after bleomycin treatment (Supplementary Fig. 12c). Gene-set enrichment analysis (GSEA) based on the Kyoto Encyclopedia of Genes and Genomes (KEGG) database further revealed that *Mfn1/2*^−/−^ AEC2 cells, compared with AEC2 cells after bleomycin treatment, markedly enhance the upregulation of purine metabolism (Supplementary Fig. 12d). Collectively, transcriptomic upregulation of purine metabolism was a common prominent feature in both *Mfn1/2*^−/−^ AEC2 cells and *Mfn1*^−/−^ and *Mfn2*^−/−^ AEC2 cells after bleomycin treatment.

### Loss of MFN1/2 alters lamellar body structure in AEC2 cells

**Fig. 7:**
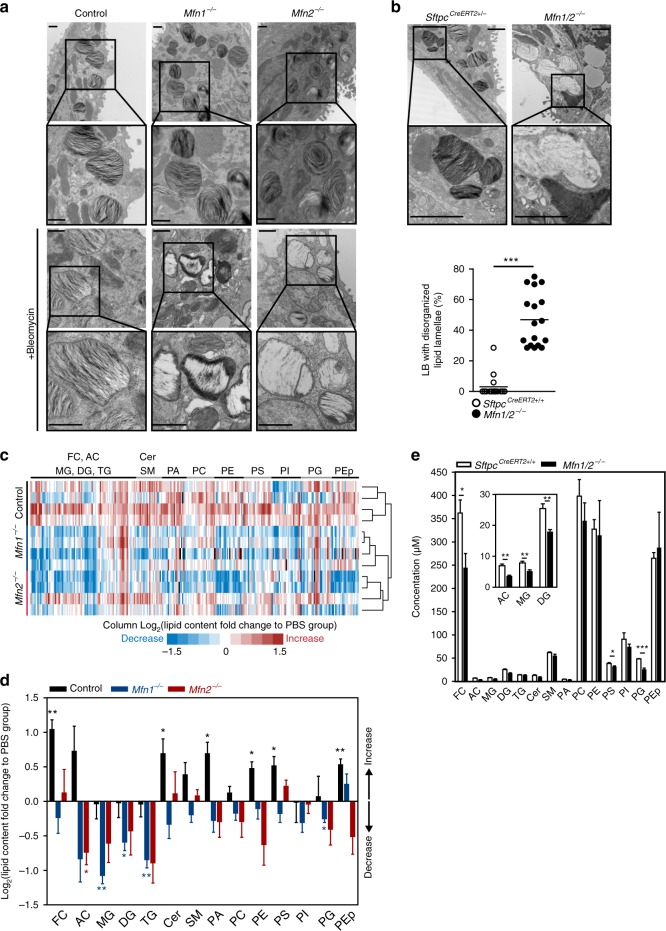
MFN1 and MFN2 regulates surfactant lipid synthesis in AEC2 cells. **a**, **b** Representative TEM images of lamellar bodies (LB) in control, *Mfn1-* and *Mfn2-*deficient AEC2 cells at baseline (×25,000; *n* = 3 mice per group) and 8 days after bleomycin treatment (×50,000; *n* = 2 mice per group) (scale bar 500 nm) (**a**) and in *Sftpc*^*CreERT2*^^+/−^ and *Mfn1/2*^−/−^ AEC2 cells (×25,000; *n* = 3 mice per group; scale bar 1 μm) (**b**, left panel). Quantification of the percentage of LB with disorganized lipid membranes in AEC2 cells by TEM image analysis (×12,000) (**b**, right panel). Each dot represents one AEC2 cell (*Sftpc*^*CreERT2*^^+/−^ AEC2 cells *n* = 15 from 3 mice, *Mfn1/2*^−/−^ AEC2 cells *n* = 17 from 2 mice; ***p < 0.001, vs. control by unpaired Student’s *t* test). **c**, **d** Heat map (**c**) and bar graph (**d**) of differential changes of specific lipid contents in control, *Mfn1*^−/−^ and *Mfn2*^−/−^ AEC2 cells (*n* = 4 biologically independent samples per group) 8 days after bleomycin treatment. The fold-changes of specific lipids in AEC2 cells after bleomycin treatment relative to those after PBS treatment (*n* = 3 biologically independent samples per group) were calculated and log-transformed (base 2) (**d**, *p < 0.05, **p < 0.01, calculated fold change vs. 1 by unpaired Student’s *t* test). **e** Lipidomic analysis in *Sftpc*^*CreERT2+/+*^ and *Mfn1/2*^−/−^ AEC2 cells (*n* = 4 mice per group; *p < 0.05, **p < 0.01, ***p < 0.001, vs. *Sftpc*^*CreERT2+/+*^ AEC2 cells by unpaired Student’s *t* test). Data are presented as mean±s.e.m. (**d**, **e**). Source data (**b**–**e**) are provided as a Source Data file

Purine synthesis and phospholipid synthesis share common upstream substrates, including serine and derivatives from glycolysis. Changes in the preferential flux of these substrates towards purine metabolism (as observed in *Mfn1*^−/−^ and *Mfn2*^−/−^ AEC2 cells after bleomycin treatment, and in *Mfn1/2*^−/−^ AEC2 cells) and away from lipid synthesis pathways (as observed in *Mfn1*^−/−^ and *Mfn2*^−/−^ AEC2 cells after bleomycin treatment) may alter the innate function of the AEC2 cells to generate phospholipids for surfactant production^32^. As previously mentioned, AEC2 cells require proper lipid metabolism to continuously produce and store (in lamellar bodies) lung surfactant, a lipoprotein complex primarily composed of lipids (90%) (particularly phosphatidylcholines and phosphatidylglycerol, and cholesterol)^4,5^. In this study we observed disrupted and disorganized lipid membranes in the lamellar bodies of *Mfn1*^−/−^ and *Mfn2*^−/−^ AEC2 cells treated with bleomycin. This was in stark contrast to control AEC2 cells treated with bleomycin or to control, *Mfn1*^−/−^ and *Mfn2*^−/−^ AEC2 cells, which all showed relatively normal lamellar body structure with organized and densely-packed lipid membranes (Fig. 7a). Unlike *Mfn1*^−/−^ or *Mfn2*^−/−^ AEC2 cells, that showed no disrupted lamellar body structure at baseline, *Mfn1/2*^−/−^ AEC2 cell lamellar bodies displayed severe disorganization of lipid lamellae (Fig. 7b), suggestive of disrupted lipid homeostasis in *Mfn1/2*^−/−^ AEC2 cells.

### *Mfn1/2* deletion impairs lipid metabolism in AEC2 cells

To evaluate if lipid metabolism was altered in AEC2 cells upon bleomycin treatment, we performed high throughput targeted lipidomic profiling in AEC2 cells of control, *Mfn1*^iΔAEC2^ and *Mfn2*^iΔAEC2^ mice at baseline and upon bleomycin treatment (8 days). At baseline, *Mfn1*^−/−^ and *Mfn2*^−/−^ AEC2 cells did not show any significant alterations in lipid species, except for a modest increase in phosphatidic acid species (Supplementary Fig. 13a-c). However, cholesterol, ceramides, phosphatidic acids, phosphatidylethanolamine, phosphatidylserine and plasmalogen phosphatidylethanolamine were all increased in AEC2 cells 8 days after bleomycin exposure (Fig. 7c, d). In contrast, these lipids were significantly decreased in *Mfn1*^−/−^ or *Mfn2*^−/−^ AEC2 cells treated with bleomycin. Specifically, acylcarnitines and phosphatidylcholines with long unsaturated aliphatic chains increased in control AEC2 cells treated with bleomycin, but not in the *Mfn1*^−/−^ or *Mfn2*^−/−^ AEC2 cells treated with bleomycin (Supplementary Fig. 14a, b). Many phosphatidylglycerol species decreased in the *Mfn1*^−/−^ AEC2 cells treated with bleomycin, but not in the control AEC2 cells treated with bleomycin (Supplementary Fig. 14c). Surfactant protein gene (*Sftpb*, *Sftpc*) expression was significantly downregulated in control, *Mfn1*^−/−^ and *Mfn2*^−/−^ AEC2 cells treated with bleomycin (Supplementary Fig. 14d). These lipid profiling results confirm that deletion of either *Mfn1* or *Mfn2* perturbs lipid metabolism in murine AEC2 cells after bleomycin treatment.

We next evaluated the lipidome of *Mfn1/2*^−/−^ AEC2 cells. Strikingly, lipidomic changes in cholesterol, acylcarnitine, monoacylglycerol, diacylglycerol, phosphatidylserine, and phosphatidylglycerol were distinctly apparent in the *Mfn1/2*^−/−^ AEC2 cells, when compared to controls (Fig. 7e). Specifically, we found that long-chain acylcarnitines significantly decreased in *Mfn1/2*^−/−^ AEC2 cells, when compared to control AEC2 cells (Supplementary Fig. 15a). Phosphatidylglycerol synthesized in mitochondria were the major phospholipid species affected in *Mfn1/2*^−/−^ AEC2 cells (Supplementary Fig. 15b). Furthermore, diacylglycerol, which is derived from phosphatidic acid, is required for the synthesis of glycerophospholipids in the ER. Several diacylglycerol species (Supplementary Fig. 15c) and certain glycerophospholipids and sphingolipids (Supplementary Fig. 15d) were all markedly decreased in the *Mfn1/2*^−/−^ AEC2 cells. Surfactant protein gene (*Sftpb*, *Sftpc*) expression was not altered between control and *Mfn1/2*^−/−^ AEC2 cells (Supplementary Fig. 15e). The above results together strongly implicate perturbed lipid metabolism in *Mfn1/2*^−/−^ AEC2 cells.

### Impaired AEC2 cell lipid synthesis promotes lung fibrosis

**Fig. 8:**
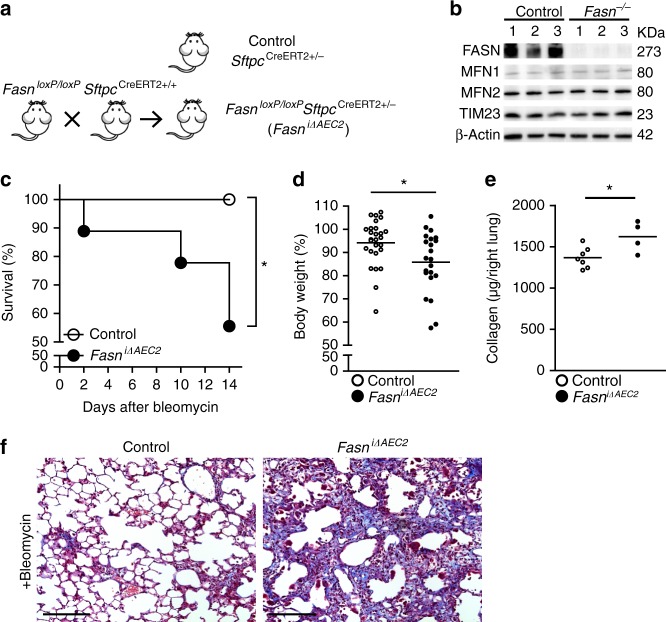
Impaired fatty acid synthesis in AEC2 cells promotes bleomycin-induced lung fibrosis. **a** Schema outlining the generation of mice with tamoxifen-inducible *Fasn* knockout in AEC2 cells. **b** Immunoblots showing MFN1, MFN2, TIM23 and β-actin expression in AEC2 cells from control or *Fasn*^iΔAEC2^ mice (*n* = 3 mice per group). **c**–**e** Kaplan–Meier survival curves (control *n* = 9, *Fasn*^iΔAEC2^ *n* = 9; *p < 0.05, by log-rank test) (**c**), body weight changes (control *n* = 27, *Fasn*^iΔAEC2^ *n* = 22) (**d**) and acid-soluble collagen depositions in right lung (control *n* = 7, *Fasn*^iΔAEC2^ *n* = 4) after bleomycin treatment (**e**) (**d**, **e** the line indicates mean; *p < 0.05, by unpaired Student’s *t* test). **f** Representative Masson’s Trichrome-stained lung sections (200X magnification; scale bar 200 μm) of mice 14 days after bleomycin treatment (*n* = 5 mice per group). Source data (**b**–**e**) are provided as a Source Data file

To test the hypothesis that loss of surfactant associated lipid metabolism in AEC2 cells contributes to the development of lung fibrosis (Supplementary Fig. 16), we compromised lipid synthesis in AEC2 cells. *FASN* encodes the principal enzyme that catalyzes the synthesis of palmitoyl-CoA, the substrate required for glycerophospholipid and sphingolipid synthesis (Supplementary Fig. 11g)^33^. We generated mice with tamoxifen-inducible *Fasn* deletion in AEC2 cells (*Fasn*^*loxP/loxP*^*Sftpc*^*CreERT2*^^+/−^, referred to as *Fasn*^iΔAEC2^), by crossing *Sftpc*^*CreERT2+/+*^ with *Fasn*^*loxP/loxp*^ mice (Fig. 8a). *Fasn* is mainly expressed in AEC2 cells^34^, and immunoblots of AEC2 cell lysates showed FASN depletion after tamoxifen injection (Fig. 8b). Notably, *Fasn*-deletion did not alter the expression levels of MFN1 and MFN2. Exposure of *Fasn*^iΔAEC2^ mice to bleomycin resulted in higher mortality (Fig. 8c), more weight loss (Fig. 8d), and increased collagen deposition and lung fibrosis (Fig. 8e, f), when compared to *Sftpc*^*CreERT2*^^+/−^ controls. These findings confirm that defective lipid metabolism in AEC2 cells promotes bleomycin-induced lung fibrosis, and supports our hypothesis that impaired regulation of lipid metabolism in AEC2 cells of *Mfn1*^iΔAEC2^, *Mfn2*^iΔAEC2^, and *Mfn1/2*^iΔAEC2^ mice contributes to development of lung fibrosis (Supplementary Fig. 16).

## Discussion

Here we uncover a critical function for MFN1 and MFN2 in AEC2 lipid metabolism and development of lung fibrosis. In the absence of mitofusins in AEC2 cells, we observe significant morbidity and mortality associated with disordered mitochondrial dynamics, including impairment of surfactant lipid metabolism and the development of spontaneous lung fibrosis.

We herein confirm that mitochondrial damage is a key pathogenic event in AEC2 cell injury and the development of lung fibrosis. Using the AEC2 cell-specific *Sftpc*-promoter, we are capable of introducing mitochondrial damage exclusively in AEC2 cells, supporting prior evidence that persistent AEC2 cell mitochondrial damage is pathogenic in the lung fibrotic process in IPF lungs and murine models^17,18^. Our findings also echo prior studies suggesting that IPF may be a single-cell disease affecting AEC2 cells^35,36^, which may in turn promote the activation of highly activated fibroblasts and myofibroblasts. Although the interaction between AEC2 cells and fibroblasts in the lung fibrotic process is not completely understood, our current work supports the present theory that injury to AEC2 cells hampers the maintenance of the epithelial cell barrier integrity. This in turn may encourage the aberrant alveolar repair process eventually leading to the extensive lung remodeling observed in IPF^35,36^.

Although it is technically difficult to access the role of mitochondrial fusion proteins MFN1 and MFN2 in regulating lipid metabolism in human AEC2 cells from healthy and IPF lungs, in this study we provide evidence that MFN1 and MFN2 regulate lipid metabolism in murine AEC2 cells, which has important ramifications for surfactant lipid production in these cells and importantly for the development of lung fibrosis. Notably, this study confirms mitochondrial fragmentation and increased synthesis of cholesterol, ceramides, and specific glycerophospholipids in response to bleomycin-induced mitochondrial damage. We believe that, in response to mitochondrial damage, AEC2 cells upregulate these lipids as an attempt to maintain surfactant lipid production under conditions of AEC2 cell injury. Loss of surfactant integrity leads to loss of normal lung physiology and may promote the development of lung fibrosis^37–39^. Most interestingly, we herein find that AEC2 cells require mitofusins to engage such lipid metabolic rewiring programs in order to respond to mitochondrial damage. We reveal that MFN1 or MFN2 deficiency abolishes the lipogenic metabolic response in AEC2 cells under bleomycin-induced mitochondrial damage and demonstrate that impaired regulation of lipid metabolism in the *Mfn1/2*^iΔAEC2^ mice drives lung fibrosis. These findings are consistent with prior findings that both human MFN1 and MFN2 are required to restore cholesterol synthesis in *Drosophila* larvae incapable of mitochondrial fusion^40^ and that both Fzo1 (the *Caenorhabditis elegans* homolog of mitofusins in mammalian cells), and lipid synthesis are required for mitochondrial stress adaptation^41^. Our findings are also consistent with mitofusins, particularly MFN2, directly regulating the interaction between the mitochondrial outer membrane and the ER^8,42^. Consistently, such distinct functions of MFN1 and MFN2 are highlighted by the observation that *Mfn2*^iΔAEC2^ mice develop more severe lung fibrosis than the *Mfn1*^iΔAEC2^ mice. In addition, the mechanisms by which *Mfn1*^iΔAEC2^ or *Mfn2*^iΔAEC2^ mice are more susceptible to bleomycin may not fully explain the development of spontaneous fibrosis in the *Mfn1/2*^iΔAEC2^ mice, with *Sftpc* expression reduced in *Mfn1*- or *Mfn2*-deficient AEC2 cells in bleomycin-induced lung fibrosis, but remaining unchanged in *Mfn1/2*-deficient AEC2 cells. The differential roles of MFN1 and MFN2 in AEC2 function regulation and lung fibrosis development require further exploration.

Intra-tracheal bleomycin administration induces acute lung inflammation and epithelial cell injury, followed by epithelial cell repair and fibrotic reactions^3,22,43^. In this study, we did not observe any difference in inflammation or altered AEC2 cell death or proliferation in *Mfn1*^iΔAEC2^ and *Mfn2*^iΔAEC2^ mice, suggesting AEC2 cell dysfunction may promote lung fibrosis independent of inflammation and cell injury. Mechanistically, data generated using *PolgA*^D257A/D257A^ mice indicates that failure of mitochondrial bioenergetics alone may not account for the phenotypes observed in the *Mfn1*^iΔAEC2^, *Mfn2*^iΔAEC2^, or *Mfn1/2*^iΔAEC2^ mice. Instead, we put forward a hypothesis whereby transcriptional alterations in key enzymes important for lipid metabolism including the fatty acid synthesis enzyme, FASN regulate lipid synthesis in AEC2 cells after bleomycin treatment. Through AEC2 cell-specific deletion of *Fasn*, we confirm that loss of lipid synthesis in AEC2 cells, upon mitochondrial damage exacerbates lung fibrosis in murine models. Similar to our findings, others have shown that perturbed long-chain fatty acid synthesis in AEC2 cells aggravates bleomycin-induced lung fibrosis^44^. Our results are also supported by findings of abnormal lipid profiles in the BALF^45^ and altered lipid synthesis in the lungs^46^ and AEC2 cells from IPF patients^20^. These findings also re-emphasize the critical role for and the proper composition of alveolar surfactant in maintaining the function and the intactness of the lung during lung injury^37,38^. Although the mechanistic link between impaired lipid synthesis in AEC2 cells and fibroblast activation requires further investigation, our data suggest that alterations in the lipidome of the lung microenvironment may promote the activation of fibroblasts and myofibroblasts. Interestingly, FASN has been identified as a TGF-β-regulated target in fibroblasts in vitro and in response to bleomycin in vivo and pharmacologically inhibiting this pathway reverses the pro-fibrotic response in the lung^47^. These findings suggest that lipid synthesis may play a distinct role in AEC2 cells and fibroblasts of the lung.

In addition to altered lipid metabolism we observed another metabolic feature of cells with mitochondrial damage^48^, namely, the upregulation of de novo purine synthesis, a common feature between *Mfn1/2*^−/−^ AEC2 cells at baseline and *Mfn1*^−/−^ or *Mfn2*^−/−^ AEC2 cells after bleomycin treatment. We hypothesize that the robust upregulation of purine metabolism in AEC2 cells may promote lung fibrosis indirectly by impairing lipid metabolism in AEC2 cells. The correct regulation of purine and lipid metabolism during mitochondrial damage is important for the diversion and utilization of common upstream substrates shared by both of these pathways, and our data indicate that mitofusins and mitochondrial fusion are essential in balancing such metabolic reprogramming. The biological significance of increased purine synthesis upregulation is not yet clear, but may occur as a compensatory mechanism to ATP synthesis when mitochondrial bioenergetic function is impaired^49^. Altered purine synthesis has been shown to promote lung inflammation and collagen deposition in murine models^50^ and inhibiting purine synthesis may offer therapeutic potential for IPF^51^. Collectively, the marked upregulation of purine metabolism in *Mfn1/2*^−/−^ AEC2 cells and in bleomycin-treated *Mfn1-* or *Mfn2-*deficient AEC2 cells may play a direct role in promoting lung fibrosis, and the pathogenic role of AEC2-specific purine metabolism warrants future investigation.

Our findings of impaired mitophagy in MFN2-deficient MLE 12 cells are consistent with previous studies demonstrating that MFN2 regulates mitophagy and autophagy^14,16,52^. However, despite the key role for MFN2 and possibly MFN1 in mitophagy regulation in AEC2 cells, *Mfn1*^iΔAEC2^ and *Mfn2*^iΔAEC2^ mice did not develop lung fibrosis, suggesting that mitofusin-related mitophagy regulation might not be the most critical mechanism accounting for lung fibrosis in the *Mfn1/2*^iΔAEC2^ mice. Currently, the cell-specific role of PINK1 and other mitophagy related pathways in the development of lung fibrosis is controversial; with some showing that global *Pink1* deficiency promotes pulmonary fibrosis^17,18^ and others demonstrating that *Pink1* deficiency in lung macrophages is anti-fibrotic^53^. Further studies into the AEC2 cell-specific role of PINK1 and mitophagy in the development of pulmonary fibrosis are required.

In conclusion, we reveal a function for MFN1 and MFN2 in mediating lung fibrosis by regulating lipid metabolism in response to mitochondrial injury in AEC2 cells of the lung, supporting the mitochondrion as a critical signaling and metabolic hub in AEC2 cells, required to modulate and control AEC2 cellular adaptation to lung fibrosis. We herein show that impaired mitochondrial damage-associated changes in lipid metabolism in AEC2 cells alter the crucial production of cholesterol and phospholipids required for surfactant synthesis and alveolar homeostasis in the lungs, and that loss of surfactant lipids as regulated by MFN1/2 disrupts the maintenance of epithelial barrier intactness and drives the fibrotic process. We thus present here the mitocentric concept that the failure of the AEC2 cell to engage in the correct metabolic and transcriptional program in response to mitochondrial damage, drives AEC2 cell injury and subsequent disordered fibrotic remodeling in the pathogenesis of lung fibrosis.

## Methods

### Mice

*Mfn1*^*loxp/loxp*^ (stock 029901-UCD) and *Mfn2*^loxp/loxp^ (stock 029902-UCD) mice were both generated by David C Chan^12^, and were purchased from Mutant Mouse Resource & Research Centers (MMRRC). *Sftpc*^CreERT2+/+^ mice were shared from Dr. Brigid Hogan^22^. *PolgA*^D257A/D257A^ mice were purchased from the Jackson Laboratory^29^. *Fasn*^loxp/loxp^ mice were kindly provided by Dr. Clay F Semenkovich, Washington University School of Medicine^33^. To generate mice with tamoxifen-inducible *Mfn1*, *Mfn2*, *Mfn1/2* or *FASN* deletion specifically in AEC2 cells, *Mfn1*^loxp/loxp^, *Mfn2*^loxp/loxp^ and *Fasn*^*loxp/loxp*^ were crossed to *Sftpc*^*CreERT2+/+*^ mice. To induce recombination by CreERT2, 6 consecutive intraperitoneal tamoxifen (100 mg/kg/dose; catalog T5648, Sigma-Aldrich) injections, prepared using sunflower seed oil (catalog S5007, Sigma-Aldrich), were given from 5 weeks of age^24^. *Sftpc*^*CreERT2*^^+/−^ or *Sftpc*^*CreERT2+/+*^ mice were used as control for experiments. Additionally, *ROSA26*^tdTomato+/+^ mice (stock 007914) were purchased from the Jackson Laboratory^54^, and were bred with *Sftpc*^CreERT2+/+^ to express tamoxifen-inducible tdTomato fluorescence in AEC2 cells^22^. To sort AEC2 cells with loss of MFN1 or MFN2 through tamoxifen-inducible tdTomato fluorescence, *ROSA26*^tdTomato+/+^ mice were crossed to *Mfn1*^loxp/loxp^ or *Mfn2*^loxp/loxp^ mice to respectively generate *Mfn1*^loxp/loxp^*ROSA26*^tdTomato+/+^ or *Mfn2*^loxp/loxp^*ROSA26*^tdTomato+/+^ mice, which were subsequently crossed to *Mfn1*^*loxp/loxp*^*Sftpc*^*CreERT2+/+*^ or *Mfn2*^loxp/loxp^*Sftpc*^CreERT2+/+^ mice. *ROSA26*^*tdTomato*^^+/−^*Sftpc*^CreERT2^^+/−^ mice were used as the control. All animal experiments and procedures in this study were approved by the Institutional Animal Care and Use Committee at Weill Cornell Medicine, and were performed in compliance with all relevant ethical regulations.

### Bleomycin model of lung fibrosis

12-week-old sex and weight matched mice were used for bleomycin instillations. Induction of anesthesia was performed in the induction chamber by 3.5% Isoflurane, and 0.5−0.75 mg/kg bleomycin (catalog 13877, Cayman Chemical Company) in 50 μL phosphate-buffered saline (PBS) was then given by intra-tracheal instillation, through gel-loading tips under the assistance of direct laryngoscopy using the otoscope^26,55^. Control mice received intra-tracheal instillation of 50 μL PBS only. The weight of mice was recorded before and every 2 days after bleomycin treatment. Mice were euthanized at different time points after bleomycin instillation for sample harvest as outlined in the manuscript and figure legends.

### Sircol assay

Murine lungs were harvested 14 days after bleomycin or PBS instillation for quantification of the acid soluble collagen, using the Sircol assay (catalog S1000, Biocolor). Murine lungs were first perfused using PBS, and the right lungs were obtained for the measurements, according to the manufacturer’s instructions.

### Bronchoalveolar lavage

After the mouse was euthanized, the trachea was intubated with a 20-gauge catheter (Terumo). Murine lungs were lavaged with 0.7-ml ice cold PBS 3 times (a total of 2.1 ml), and the BALF was collected. After centrifugation at 500 × *g* for 5 min at 4 °C, the supernatant was aliquoted and stored at −80 °C; BALF protein concentration was measured using BCA protein assay kit (Thermo Fisher). Cell pellets were re-suspended in 100 μL PBS. The cell number was quantified using 10 μL cell suspension by a Countess II Automated Cell Counter (Thermo Fisher), and cytospin slides were prepared using 40 μL of the cell suspension with 160 μL of PBS [28.23 × *g* (500 r.p.m.) for 5 min]. Slides were stained using the Hemacolor Rapid staining kit (EMD Millipore), and the numbers of macrophages, leukocytes and neutrophils were counted in a total of at least 200 cells.

### Isolation of murine AEC2 cells through MACS separation

AEC2 cells were isolated from murine lungs^23^. Mice were euthanized by intraperitoneal injection of 8 mg pentobarbital, and a thoracotomy was performed. Murine lungs were perfused through the right ventricle using PBS, and then inflated with 1.5 mL dispase (catalog 354235, BD Biosciences) and 0.5 mL 1% low-melting point agarose (catalog 16520–050, Invitrogen). After cooling on ice for 2 min, the lungs were excised and were transferred to a 50 ml polypropylene tube containing 2 mL dispase. After digestion for 45 min at room temperature, the lungs were homogenized manually using the plunger of a 1 mL syringe in a 10 cm petri dish with Dulbecco’s modified Eagle’s medium (DMEM) containing 200 U/mL DNase (catalog D-4527, Sigma-Aldrich). After filtration sequentially through 100, 40 μm (BD Biosciences), and 0.22 μm (EMD Millipore) strainers, and centrifugation (200 × *g* at 4 °C for 10 min), the supernatant was discarded and the cell pellets were then suspended in MACS buffer (90 μL/10^7^ cells). MACS buffer composes of PBS containing 0.5% bovine serum albumin (catalog 130–091–376, Miltenyi Biotec) and 2 mM EDTA (AM9260G, Invitrogen). The cell suspensions were then negatively selected for anti-mouse CD45 microbeads (10 μL/10^7^ cells; catalog 130–052–301, Miltenyi Biotec), followed by positive selection with biotin-conjugated anti-mouse EpCAM antibody (1:150; catalog 13–5791–82, eBioscience), streptavidin microbeads (10 μL/10^7^ cells; catalog 130–048–102, Miltenyi Biotec) and anti-mouse FcR blocking reagent (10 μL/10^7^ cells; catalog 130–092–575, Miltenyi Biotec), through MACS separation columns. All the reagents for cell selection were diluted using MACS buffer. The resulting CD45(-)EpCAM(+) population was enriched for AEC2 cells (purity ~94% by flow cytometric analysis and quantification by immunofluorescence staining; please *see* flow cytometric and immunofluorescence staining methods below).

### Immunofluorescent staining of isolated AEC2 Cells

To quantify the AEC2 purity in MACS-isolated CD45(-)EpCAM(+) populations, we performed immunofluorescent staining of surfactant protein C (SP-C) using cytospin slides. After isolation, the CD45(-)EpCAM(+) cells were fixed by 4% PFA in a flow cytometry tube for 12 min at room temperature, and were transferred to slides by cytospin centrifugation at 13.83 × *g* (350 r.p.m.) for 3 min. The CD45(−)EpCAM(−) population was used to prepare cytospin slides for negative control. Blocking and permeabilization were performed at room temperature for 1 h, using a buffer containing 5% normal goat serum (Vector Laboratories) and 0.3% Triton X-100 (Sigma-Aldrich) in tris-buffered saline (TBS). Cells were incubated overnight with a primary antibody generated to SP-C (1:1000 in blocking buffer, EMD Millipore ABC99) in a humidified chamber at 4 °C. Sixteen to 24 h later, the cells were incubated with the Alexa Fluor-488-conjugated secondary antibody (Thermo Fisher) for 1 h at room temperature. Hoechst 33342 (1:1000 dilution in TBS) was used to stain the nucleus. The slides were mounted using Prolong Gold antifade solution (Invitrogen), and the images of the slides were obtained by confocal microscopy (Zeiss LSM 880 laser scanning microscope).

### Flow cytometry analysis

Flow cytometric analyses of EpCAM or SP-C positivity were performed using a LSRFortessa cell analyzer (BD Biosciences). For the staining of EpCAM, cells were fixed by 1% PFA for 15 min at room temperature, followed by EpCAM binding with biotin-conjugated anti-EpCAM (1:50; eBioscience) and anti-mouse FcR blocking reagent (1:10; catalog 130–092–575, Miltenyi Biotec) for 1 h on ice. After washing, a FITC-conjugated anti-biotin antibody (1:10; catalog 130–098–796, Miltenyi Biotec) was added for 10 min on ice in the dark. After further washing, the samples were used for flow cytometric analyses. SP-C intracellular staining for flow cytometric analysis was the same as the protocol for immunofluorescent SP-C staining using cytospin slides. mtKeima experiments were carried out to assess mitophagy induction. To induce mitophagy, cells with mtKeima expression were treated with the combination of oligomycin and antimycin A (4 μM/5 μM; Sigma-Aldrich) for 24 h. Mitophagy measurement was performed by the pH-sensitive mtKeima fluorescence by the excitation using 405 nm (for detecting mtKeima at pH 7.0) and 561 nm (for detecting acidic mtKeima at pH 4.0) lasers^25,56,57^. The intensity of mitophagy was calculated by the ratio of cell percentage with acidic mtKeima (upper gate) to cell percentage with neural mtKeima (lower gate) (see also Supplementary Fig. 3h). The flow cytometric data were analyzed with FlowJo analytical software (version 10) (https://www.flowjo.com./) (BD Biosciences).

### AEC2 cell isolation by tdTomato fluorescence

To isolate AEC2 cells through tdTomato fluorescence, whole lung cell suspension was obtained after digestion and homogenization of mouse lungs, as described for AEC2 cell isolation by MACS separation. DAPI (0.1 μg/mL) was added to assess cell viability. Flow cytometric cell sorting was then performed by an Influx cell sorter (BD Biosciences) (see also Supplementary Fig. 1a).

### Genotyping for *Mfn1* deletion in AEC2 cells

DNA samples were extracted from AEC2 cells obtained from *Sftpc*^*CreERT2*^^+/−^, *Mfn1*^iΔAEC2^, *Mfn1/2*^iΔAEC2^, control^tdTomato-AEC2^, and *Mfn1*^iΔAEC2/tdTomato-AEC2^ mice using DNeasy blood and tissue kit (Qiagen), and were used for genotyping through PCR reactions and the subsequent resolution by agarose gel electrophoresis, based on the protocol by MMRRC (forward 5′-TTGGTAATCTTTAGCGGTGGTC-3′, reverse 5′-TTAAAGACACGGCTAATGGCAG-3′).

### Real-time qPCR for quantification of mtDNA copy number

DNA samples were extracted from isolated AEC2 cells, and were used for the real-time qPCR, using primers for *Nd2* gene of mitochondrial genome (forward 5′-CTATCACCCTTGCCATCAT-3′, reverse 5′-GAGGCT-GTTGCTTGTGTGAC-3′) and *Pecam* gene of nuclear genome (forward 5′-ATGGAAAGCCTGCCATCATG-3′, reverse 5′-TCCTTGTTGTTCAGCATCAC-3′)^13^ and SYBR green PCR master mix (Applied Biosystems), in the ABI PRISM 7500 Real-Time PCR System (Applied Biosystems). mtDNA copy number was calculated relative to genomic DNA (gDNA) copy number, through the 2^-ΔΔCt^ method.

### Cell lines

The murine AEC2 cell line MLE 12 and human AEC2 cell line A549 were purchased from ATCC (CRL-2100 and CCL-185, respectively), and were maintained in RPMI 1640 medium containing 10% FBS and 1% penicillin-streptomycin (Gibco). For stable knockdown of *Mfn1* or *Mfn2* in MLE 12 cells, independent small hairpin RNA (shRNA) targeting of *Mfn1* (TRCN0000081398, TRCN0000081401, and TRCN0000081402; Sigma-Aldrich) or *Mfn2* (TRCN0000080610, TRCN0000080611 and TRCN0000080612; Sigma-Aldrich) were used, and non-target shRNA (SHC016; Sigma-Aldrich) served as the control. MLE 12 cells were transduced by shRNA lentiviral particles, followed by puromycin (2 μg/mL; catalog A11138–03, Gibco) positive selection for 10–14 days, and were then maintained in RPMI 1640 medium containing 2 μg/mL puromycin and 0.5% penicillin-streptomycin. Retroviral packaging plasmids were gifts from David C Chan. The retroviral construct pCHAC-mt-mKeima was a gift from Richard Youle (Addgene plasmid #72342)^25,56^, and was used to express mtKeima in MLE 12 cells through retroviral transduction. Cell sorting by Influx sorter (BD Biosciences) was performed to obtain mtKeima-positive cells. Based on manufacturer’s instructions, A549 cells were transfected with non-targeting control siRNA (Dharmacon, D-001206-14-05) or siRNA targeting at human *Mfn1* (Dharmacon SMARTpool, M-010670-01-0005) or *Mfn2* (Dharmacon SMARTpool, M-012961-00-0005) mRNA using Lipofectamine® RNAiMAX Transfection Reagent (Life Technologies). The above cell lines were free of mycoplasma infection, assessed using EZ-PCR^TM^ Mycoplasma detection kit (Biological Industries).

### Immunoblots

Immunoblotting was performed using lysates of MLE 12 or MACS®-isolated AEC2 cells. RIPA buffer with protease inhibitor cocktail (Cell Signaling Technology) was used to prepare the lysates, and the protein concentrations were measured using BCA protein assay (Thermo Fisher). Proteins were resolved by NuPAGE 4–12% Bis-Tris gel or 3–8% Tris-Acetate gel (Invitrogen) electrophoresis, followed by transfer to PVDF membranes (EMD Millipore). For immunoblots using A549 lysates, proteins were resolved using 8% Tris-glycine gels. The following primary antibodies were used to detect murine MFN1 (1:1000, Antibodies Incorporated 75–162), human MFN1 (1:1000, Proteintech 13798-1-AP), MFN2 (1:1000, Cell Signaling Technology 9482), OPA1 (1:1000, GeneTex GTX48589), DRP1 (1:500, BD Biosciences, 611112), FASN (1:1000, Cell Signaling Technology 3180), TIM23 (1:1000, BD Biosciences 611223) and β-Actin (1:5000, Sigma-Aldrich A2228). The horseradish peroxidase (HRP)-conjugated secondary antibodies, anti-rabbit IgG (1:5000, Santa Cruz sc-2004 or GeneTex 213110) and anti-mouse IgG (1:5000, Santa Cruz sc-2005 or BioLegend 405306), were used. Densitometric quantification of bands was carried out using FIJI running ImageJ software (version 1.52b) (https://fiji.sc/), normalizing to β-actin as a loading control. All the full blots can be found online in the Source Data file.

### Lung histology and immunohistochemistry (IHC) staining

For histological examination, murine lungs were inflated by 1.2 mL 4% paraformaldehyde (PFA) (Electron Microscopy Sciences), and then transferred to a 50-ml polypropylene tube containing 10 mL 4% PFA. After 24-h of fixation at 4 °C, the lobes of the mouse lungs were separated, and transferred to tissue cassettes (Tissue-Tek). The tissue cassettes were then immersed in 70% ethanol at 4 °C. Primary antibodies against vimentin (1:100, Cell Signaling Technology, 5741), α-smooth muscle actin (1:640, Cell Signaling Technology, 19245), and collagen III (1:1000, Abcam, ab7778) were used for IHC staining. For IHC staining, the paraffin-embedded lung sections were first baked and deparaffinized. To retrieve antigen, the slides were heated on the Bond III Autostainer at 99–100 °C, and the sections subjected to sequential incubation with an endogenous peroxidase block, primary antibody, secondary antibody, polymer, diaminobezidine, and hematoxylin. Finally, the sections were dehydrated in 100% ethanol, and mounted in Cytoseal XYL (Richard Allan Scientific). Appropriate positive and negative controls were included.

### Immunofluorescent staining of mouse lung cryosections

To prepare lung cryosections for immunofluorescent staining, murine lungs were inflated using 1.2 mL 4% PFA. The lungs were then excised and transferred to a 50 mL polypropylene tube containing 10 mL 4% PFA for 24-hour fixation at 4 °C. After fixation, the lobes were separated, and transferred to 30% sucrose (Sigma-Aldrich) solution for 24 h at 4 °C. Thereafter, the lung lobes were placed in a cryomold (Tissue-Tek), and embedded by optimum cutting temperature (OCT) formulations. The samples were then stored at −80 °C, and 15-μm-thick cryosections were retrieved on silane-coated slides immediately before immunofluorescent staining. Blocking and permeation of the cryosections was performed using TBS buffer containing 5% normal donkey serum (Jackson ImmunoResearch) and 0.3% Triton X-100 (Sigma-Aldrich). Primary antibodies against SP-C (1:1000, EMD Millipore ABC99), podoplanin (1:100, R&D Systems AF3244), and Ki-67 (1:500, Abcam, ab15580), and secondary antibodies against goat IgG (linked to Alexa Fluor-488) or rabbit IgG (linked to Alexa Fluor-488 or Alexa Fluor-568) (1:500, Thermo Fisher), were all diluted in blocking buffer, and were used for staining the cryosections. A primary antibody against ER-TR7 was conjugated with Alexa Fluor-647, and was used to stain fibroblasts (1:50, Santa Cruz sc-73355 AF647). Cryosections were covered with diluted primary antibodies and incubated in a humidified chamber overnight at 4 °C. Sixteen to 24 h later, the cryosections were incubated with secondary antibodies for 1 h under room temperature, with protection from light exposure. Hoechst 33342 (1:1000 dilution in TBS) was used to stain the nucleus. The slides were mounted using Prolong Gold antifade solution (Invitrogen), and the images of the slides were obtained by confocal microscopy.

### TUNEL staining

Mouse lung cryosections were used for TUNEL staining by ApoAlert^TM^ DNA fragmentation assay kit (Clontech), according to the manufacturer’s instructions with some modifications. Cryosections were permeabilized with PBS containing 0.2% Triton X-100 at 4 °C for 5 min. The samples were then covered with the equilibration buffer for 10 min at room temperature. The TdT incubation buffer was prepared according to the manufacturer’s protocol, and was added onto the samples. The slides were then placed in a humidified chamber with light protection, and were incubated at 37 °C for 1 h. SSC (saline-sodium citrate) solution (2X) was then used to immerse the slides at room temperature for 15 min, and Hoechst 33342 (1:1000) was used to stain the nucleus. The slides were mounted using Prolong Gold antifade solution (Invitrogen), and the images were obtained by confocal microscopy.

### Confocal microscopy

Confocal microscopy was used to obtain images of immunofluorescent staining, and for the visualization of mitochondria in MLE 12 and A549 cells. A Zeiss LSM 880 laser scanning microscope equipped with ×25/0.8, ×40/1.3, and ×63/1.4 oil immersion objectives was used for image acquisition. The fluorophores were excited with a 405 nm laser diode (Hoechst 33342), a 488 nm argon laser (Alexa Fluor-488), a 561 nm diode-pumped solid-state laser (Alexa Fluor-568), or a 633 nm HeNe laser (Alexa Fluor-647). To observe mitochondrial morphology in MLE 12 and A549 cells, cells were cultured in glass-bottom dishes (MatTek Corporation). Mitochondria were stained by 200 nM MitoTracker Green (Invitrogen) at 37 °C for 30 min. DMEM medium free of phenol red was used to wash the cells and for maintaining the cells for live-cell imaging. The fluorescence of MitoTracker Green was excited by a 488 nm argon laser. Tubular mitochondria were considered when mitochondria were not in spherical shape, and cells with <50% tubular mitochondria were determined when less than half of the mitochondria were in tubular shape^58^.

### Transmission Electron Microscopy (TEM)

Fixatives for TEM sample preparation were composed of 4% paraformaldehyde, 2.5% glutaraldehyde, 0.02% picric acid in 0.1 M sodium cacodylate buffer (pH 7.3). Murine lungs were inflated with 1.2 mL TEM fixative, and were then excised and transferred to a 50 mL polypropylene tube containing 10 mL TEM fixative, and were submitted to the WCMC Microscopy and Image Analysis Core Facility for sample processing and image acquisition. A Jeol electron microscope (JEM-1400) was used to obtain images with an accelerating voltage of 100 kV. AEC2 cells were identified according to the appearance of lamellar bodies and the microvilli at the apical cell membrane. The quantification of the number (#/μm^2^ cytosolic area) and the area (μm^2^) of mitochondria or lamellar bodies was performed using FIJI running ImageJ software.

### RNA-Seq analysis in AEC2 cells

RNA samples were obtained from MACS-isolated AEC2 cells from *Sftpc*^CreERT2+/+^ and *Mfn1/2*^iΔAEC2^ mice, or from AEC2 cells isolated by tdTomato(+) cell sorting from control^tdTomato-AEC2^, *Mfn1*^iΔAEC2/tdTomato-AEC2^, and *Mfn2*^iΔAEC2/tdTomato-AEC2^ mice. RNA was extracted using TRIzol reagent (Invitrogen), and was purified by the RNeasy Plus Mini Kit (Qiagen), together with DNA digestion with the RNA free DNase set (Qiagen). The RNA samples were then submitted to Genomic Resource Core Facility of WCMC. RNA quality was determined by 260:280 ratio and the RNA integrity number (RIN) determined by an Agilent Technologies 2100 Bioanalyzer. Only high quality RNA samples with a 260:280 ratio > 1.6 and a RIN > 7 were used for the library construction using the TruSeq Stranded mRNA Library Preparation kit (Illumina), according to manufacturer’s instructions. The cBot fluidic device (Illumina) was used to hybridize samples onto a flow cell and to generate cloncal clusters of the DNA fragments. The sequencing was performed on the HiSeq4000 sequencer (Illumina). The raw sequencing reads in binary base call (BCL) format were processed through bcl2fastq 2.19 (Illumina) for FASTQ format conversion and demultiplexing. RNA reads were aligned and mapped to the mm9 mouse reference genome by TopHAEC2 (version 2.0.11) (https://ccb.jhu.edu/software/tophat/index.shtml)^59^, and transcriptome reconstruction was performed by Cufflinks (version 2.1.1) (https://cole-trapnell-lab.github.io/cufflinks/), with gene names based on National Center for Biotechnology Information (NCBI) Entrez Gene. The abundance of transcripts was measured with Cufflinks in Fragments Per Kilobase of exon model per Million mapped reads (FPKM)^60,61^. Differentially expressed genes were identified using the Limma package (http://bioconductor.org/packages/release/bioc/html/limma.html)^62^. To assess the differential expression, p-values were derived from linear modelling and empirical Bayes moderation and adjusted for multiple testing by the Benjamini-Hochberg method. Gene ontology (GO) over-representation analysis was performed using the clusterProfiler package (http://bioconductor.org/packages/release/bioc/html/clusterProfiler.html)^63^. GO terms related to biological process were used, and adjusted p-values for multiple testing were calculated based on the Benjamini-Hochberg method. The over-representative GO terms were constructed into a enrichment map^64^ where the geometric mean of the Jaccard and overlap coefficients between GO-associated gene sets was used at a cutoff of 0.5 to connect related GO terms. The enrichment maps were visualized by Cytoscape (version 3.6.1), and the functional clusters were highlighted and labeled manually. Heat maps were plotted using Heatmap Illustrator software (Heml 1.0) (hemi.biocuckoo.org)^65^ and the pheatmap package (https://github.com/raivokolde/pheatmap), based on the z scores calculated using the gene expressions by FPKM.

### Lipidomic profiling in AEC2 cells

MACS-isolated AEC2 cells from 1 to 2 mice formed one sample for lipidomic analyses. AEC2 cells were snap frozen by liquid nitrogen immediately after isolation, and were stored at −80 °C before lipidomic profiling. Samples were submitted to the Columbia University Lipidomics Core Laboratroy^66,67^. Lipids were extracted from equal amounts of material (50 μg protein/sample). Lipid extracts were prepared using a modified Bligh and Dyer procedure^68,69^, spiked with appropriate internal standards, and analyzed using a 6490 Triple Quadrupole LC/MS system (Agilent Technologies). Glycerophospholipids and sphingolipids were separated with normal-phase HPLC as described before^69^, with a few modifications. An Agilent Zorbax Rx-Sil column (inner diameter 2.1 × 100 mm) was used under the following conditions: mobile phase A (chloroform:methanol:1 M ammonium hydroxide, 89.9:10:0.1, v/v) and mobile phase B (chloroform:methanol:water:ammonium hydroxide, 55:39.9:5:0.1, v/v); 95% A for 2 min, linear gradient to 30% A over 18 min and held for 3 min, and linear gradient to 95% A over 2 min and held for 6 min. Sterols and glycerolipids were separated with reverse-phase HPLC using an isocratic mobile phase as before^69^ except with an Agilent Zorbax Eclipse XDB-C18 column (4.6 × 100 mm). Quantification of lipid species was accomplished using multiple reaction monitoring (MRM) transitions that were developed in earlier studies^69^ in conjunction with referencing of appropriate internal standards: PA 14:0/14:0, PC 14:0/14:0, PE 14:0/14:0, PI 12:0/13:0, PS 14:0/14:0, SM d18:1/12:0, D_7_-cholesterol, CE 17:0, MG 17:0, 4ME 16:0 diether DG, D_5_-TG 16:0/18:0/16:0 (Avanti Polar Lipids, Alabaster, AL). Lipid levels for each sample were calculated relative to the spiked internal standards. For lipidomic analysis in AEC2 cells in the bleomycin model, the fold changes of the lipid levels in AEC2 cells after bleomycin treatment were calculated relative to the levels in AEC2 cells after PBS treatment, and were log_2_-transformed. The heatmap was plotted based on the log_2_(fold change), using Heatmap Illustrator software (Heml 1.0)^65^.

### Statistical analysis

Data are represented as outlined in the figure legends. Unpaired Student’s t-test was used for the comparisons between two groups, and one-way ANOVA with post-hoc Bonferroni test was used for multi-group comparisons. The log-rank test was used to compare the differences of the survival between two groups. The detailed statistical analyses for RNA-seq data were described in the method details of RNA-seq. A two-sided *p* value <0.05 was statistically significant. All analyses were performed using SPSS version 17.0 (IBM Corporation) or GraphPad Prism version 5.0 (GraphPad Software).

### Reporting Summary

Further information on research design is available in the Nature Research Reporting Summary linked to this article.

## Supplementary information

Supplementary Information

Description of Additional Supplementary Files

Supplementary Data 1

Supplementary Data 2

Supplementary Data 3

Supplementary Data 4

Reporting Summary

Source Data File

## Data Availability

RNA-seq data have been deposited in Gene Expression Omnibus (GEO) under the accession code GSE115730. All data and methods relevant to the findings of this study are available from the corresponding authors at request. All the TEM and confocal microscopy images in the figures and supplementary figures have been deposited in Mendeley online data repository (doi:10.17632/29v5w97mx4.3). The source data underlying Figs. 1c, d, 2c, e, f, 3c–e, 4a, b, d, 5c, e, 7b–e, 8b–e, and Supplementary Fig. 1c–e, 2c, d, 4a–c, f, 5g, 6a–c, 7a–d, 10c and 11b, d are provided as the Source Data file.
