# Supplementary material for: Mitofusins regulate lipid metabolism to mediate the development of lung fibrosis
Source: Nat Commun. 2019 Jul 29;10:3390. doi: 10.1038/s41467-019-11327-1 (PMC6662701; doi:10.1038/s41467-019-11327-1)
Supplement: Supplementary file 1 — Supplementary Information [file 41467_2019_11327_MOESM1_ESM.pdf]

SUPPLEMENTARY INFORMATION
Mitofusins Regulate Lipid Metabolism to Mediate the Development of Lung fibrosis

Kuei-Pin Chung et al.

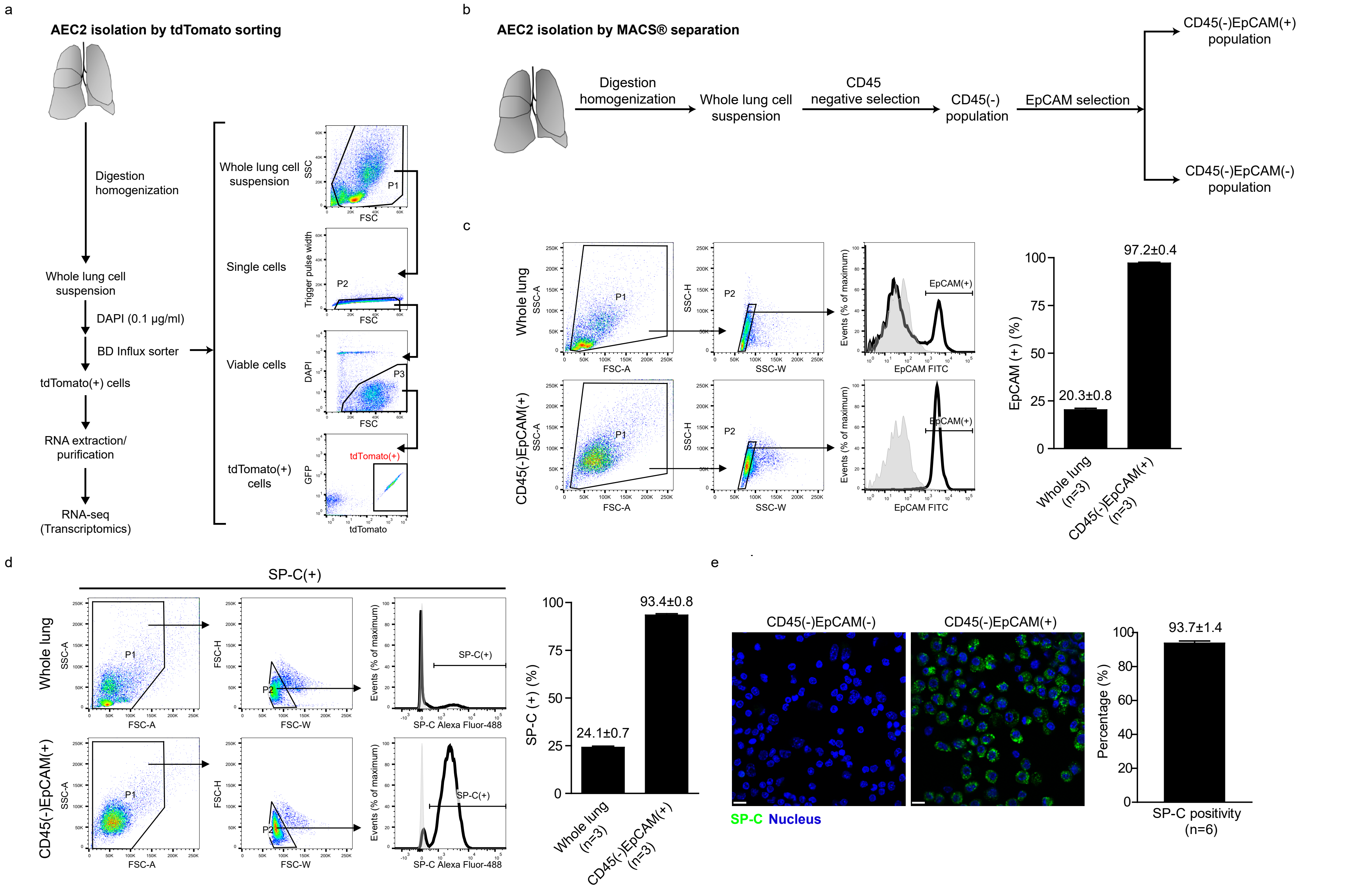

Supplementary Figure 1 Isolation of AEC2 cells from murine lungs. **a** Representative flow cytometry protocol and gating strategy to isolate tdTomato(+) cells from whole lung cell suspensions, with DAPI staining to exclude non-viable cells. **b-e** Isolation of AEC2 cells using magnetic-activated cell sorting (MACS). Schema demonstrating MACS AEC2 cell isolation protocol using CD45 negative selection and EpCAM positive selection (**b**). Purity of AEC2 cells isolated by MACS (**c-e**). Representative flow cytometric analysis and gating strategy of EpCAM (**c**) and SP-C (**d**) positive cells in whole lung cell suspensions (upper left panel) and CD45(-)EpCAM(+) cells (lower left panel) with the percentage of positive cells quantified (right panel) in the respective populations (n=3 mice per group; data are mean±s.e.m.). Representative confocal images of SP-C positive cells by immunofluorescence staining (**e**) of CD45(-)EpCAM(-) cells (negative control, left panel) or CD45(-)EpCAM(+) cells (middle panel), with quantification of the percentage of SP-C (+) cells (right panel; data are mean±s.e.m.; n=6 mice, and 3 high-powered fields are obtained per mice by confocal microscopy using a 63X/1.4 oil immersion objective; scale bar 10 µm). Source data (**c-e**) are provided as a Source Data file.

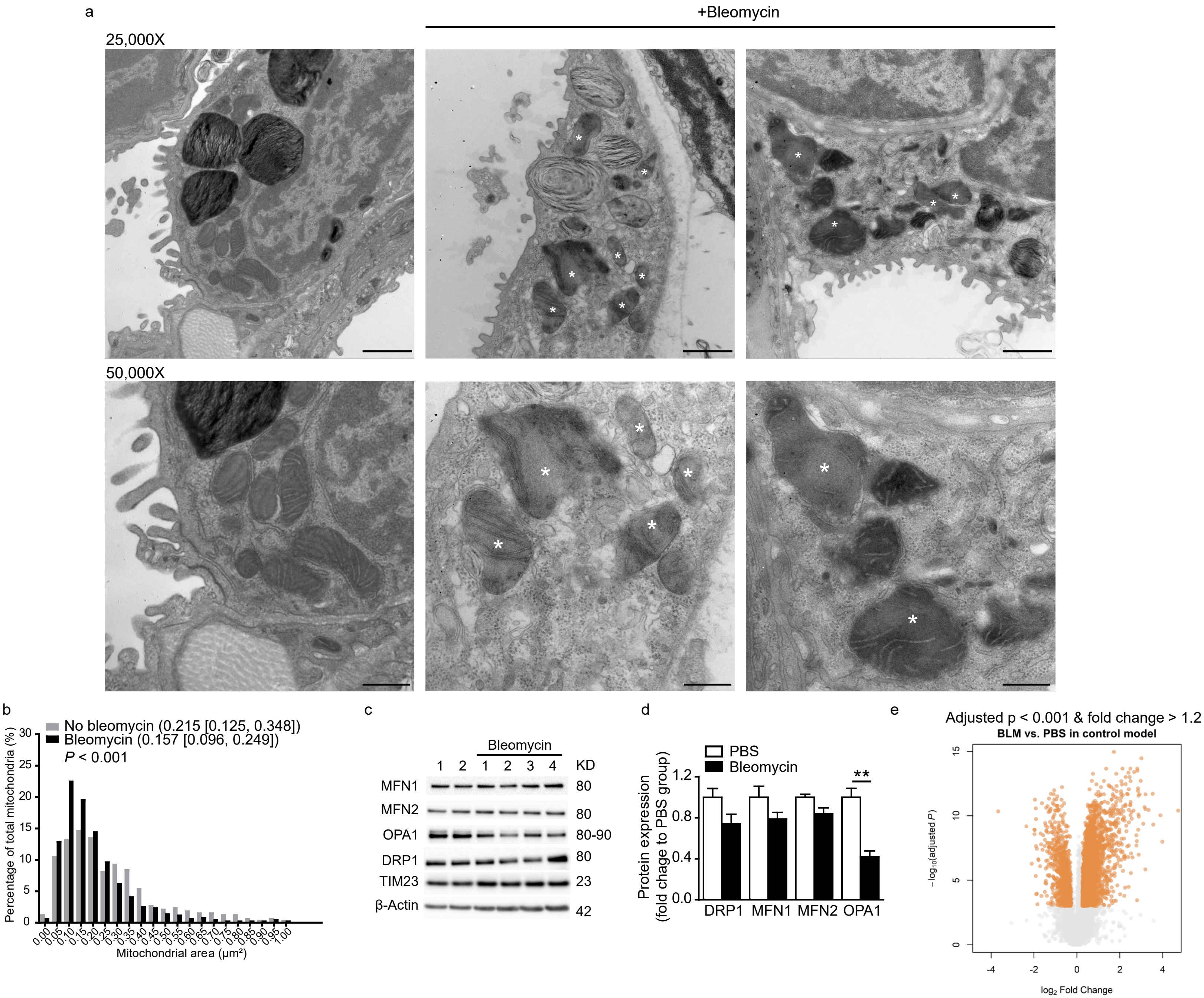

Supplementary Figure 2 Characterization of bleomycin-induced mitochondrial damage in AEC2 cells. **a** Representative TEM images (scale bar, upper panel 1 μm, lower panel 500 nm) highlighting swollen mitochondria with regional decreased electron density and disrupted cristae (marked by asterisk) in AEC2 cells after bleomycin treatment (before bleomycin, n=3 mice; after bleomycin, n=2 mice). **b** Quantification of mitochondrial area of each mitochondrion in AEC2 cells before and after bleomycin treatment. Data presented are median [interquartile range], and the comparison is performed by Mann-Whitney U test (no bleomycin n=335 mitochondria, after bleomycin n=521 mitochondria). **c, d** Immunoblots showing the expression of MFN1, MFN2, OPA1, DRP1, TIM23, and β-actin in AEC2 cell lysates 8 days after PBS or bleomcin treatment (PBS, n = 2 mice; bleomycin, n = 4 mice) (**c**), and the quantification by densitometric analysis through normalization to β-actin, expressed as the fold change relative to control (data are mean±s.e.m., ** p < 0.01 vs control, by unpaired Student's t-test) (**d**). **e** Volcano plot differently expressed genes between AEC2 cells isolated from mice treated with bleomycin (BLM) and from mice treated with PBS control; threshold of an adjusted p < 0.001 and a fold change > 1.2. Source data (**b-d**) are provided as a Source Data file.

a

Mfn2$^{-/-}$ AEC2 cells

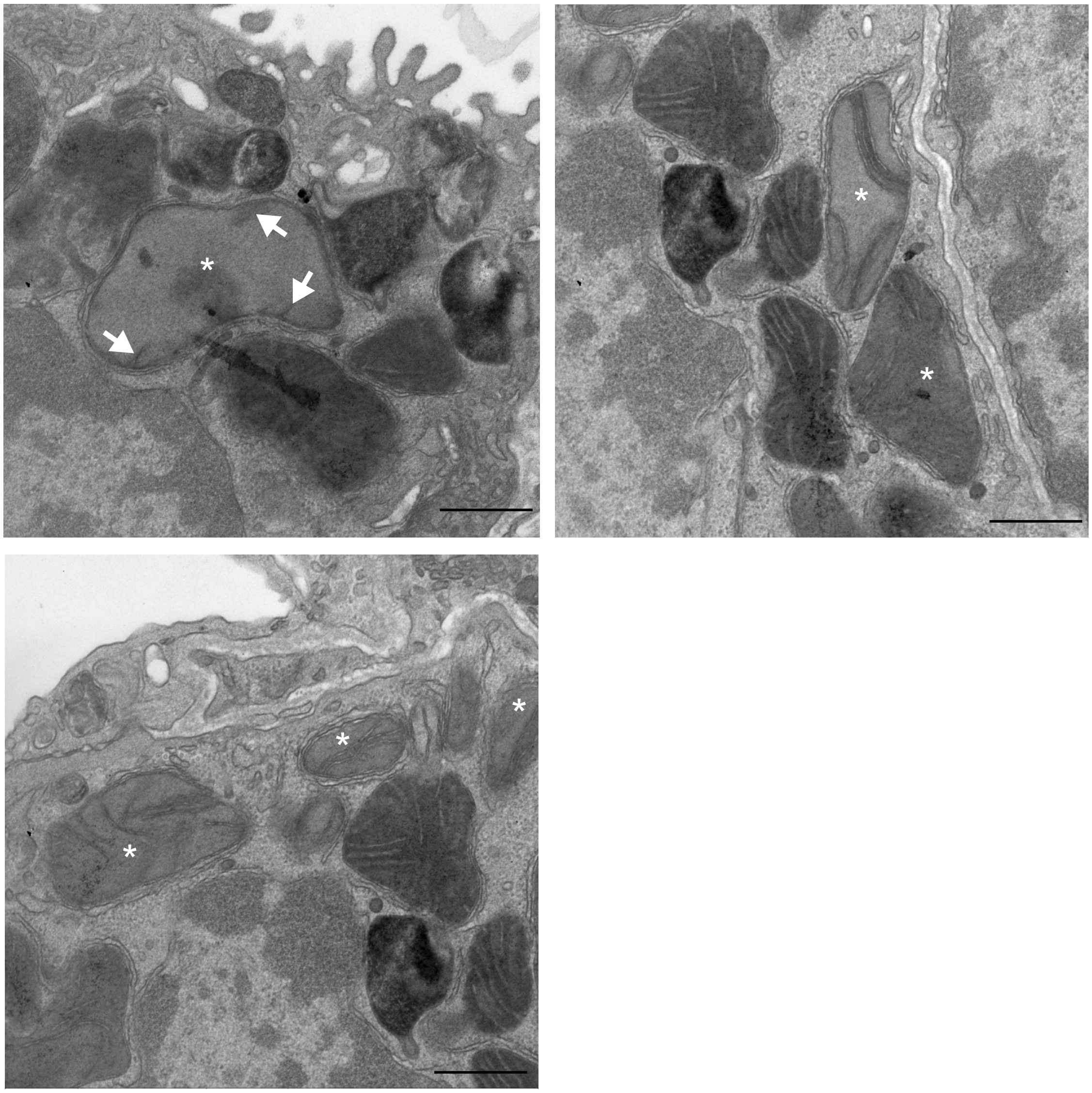

b

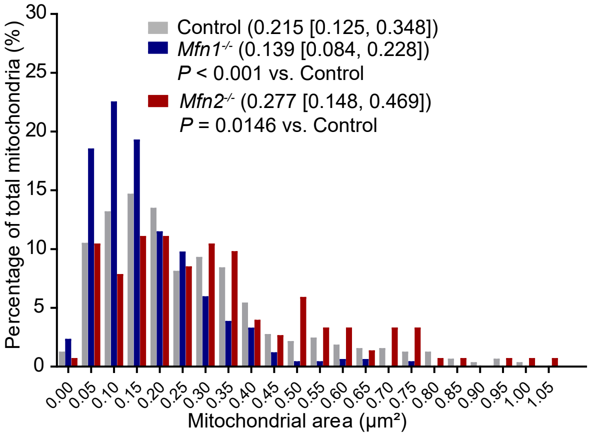

Supplementary Figure 3 MFN1 and MFN2 regulate mitochondrial morphology in murine AEC2 cells. **a** Representative TEM images (50,000X; scale bar 500 nm) highlighting two distinct mitochondrial morphologies in *Mfn2*$^{-/-}$ AEC2 cells; 1) relatively normal mitochondria with enlarged size but regular cristae, and 2) abnormal mitochondria with disrupted and irregular cristae (marked by asterisk). The arrows point to the residual cristae in a swollen mitochondrion (upper panel, left image). **b** Quantification of mitochondrial area of each mitochondrion in AEC2 cells. Data presented are median [interquartile range], with comparison by Mann-Whitney U test (control n=335 mitochondria; *Mfn1*$^{-/-}$ n=525 mitochondria; *Mfn2*$^{-/-}$ n=154 mitochondria). Source data (**b**) are provided as a Source Data file.

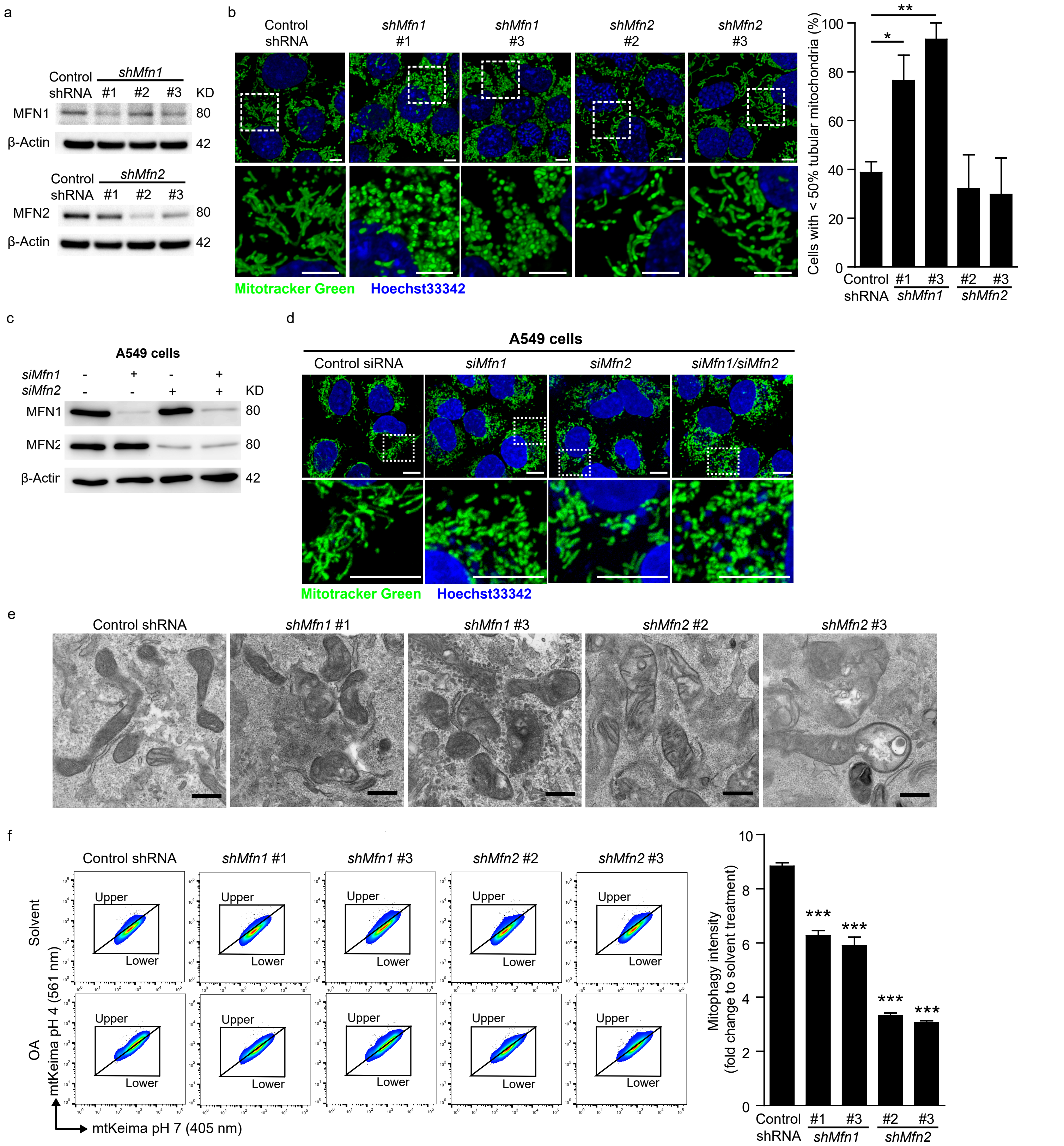

Supplementary Figure 4 MFN1 and MFN2 regulate mitochondrial morphology in murine (MLE 12) and human (A549) AEC2 cell lines. **a** Immunoblots for MFN1 (upper panel) and MFN2 (lower panel) expression in *Mfn1*- or *Mfn2*- depleted MLE 12 cells generated using shRNA lentiviral transduction (n=3 technical repeats). **b** MLE 12 cells were infected with shRNA targeted to MFN1 or MFN2 and the mitochondrial fluorescent dye MitoTracker green was used to label mitochondria (left panel; scale bar 5 μm). Lines with higher knockdown efficiency were selected for live-cell confocal imaging (a 3-dimensional reconstruction image from 2.46-μm-thick z stacks through a 63X/1.4 oil immersion objective) and the percentages of cells with < 50% tubular mitochondria were quantified (right panel). Three high power fields were randomly selected, and cells in each high power field were quantified. Data are mean±s.e.m. (*p < 0.05, **p < 0.01, vs. control by unpaired Student's t-test). **c, d** Immunoblots for *Mfn1* and *Mfn2* siRNA knockdown efficacy (**c**), and representative mitochondrial morphology (live-cell confocal images of MitoTracker green staining, 3-dimensional reconstruction images from 2-μm-thick z stacks through a 63X/1.4 oil immersion objective) in the human AEC2 cell line A549 (n=5 random-selected fields for each condition; scale bar 10 μm) (**d**). **e** Representative TEM images (50,000X) of mitochondria in MLE 12 cells (n=5 random-selected fields under 5,000X per cell line; scale bar 500 nm). **f** Representative flow cytometric analysis of mitophagy induced by oligomycin/antimycin (OA) in MLE 12 cells using mtKeima (left panel). Mitophagy intensity was quantified based on the cell percentage in the upper gate, and the fold change was calculated relative to the solvent group (right panel). Data are mean±s.e.m. of triplicates (n=2 technical repeats; ***p < 0.001, vs control by unpaired Student's t-test). Source data (**a**, **b**, **c**, **f**) are provided as a Source Data file.

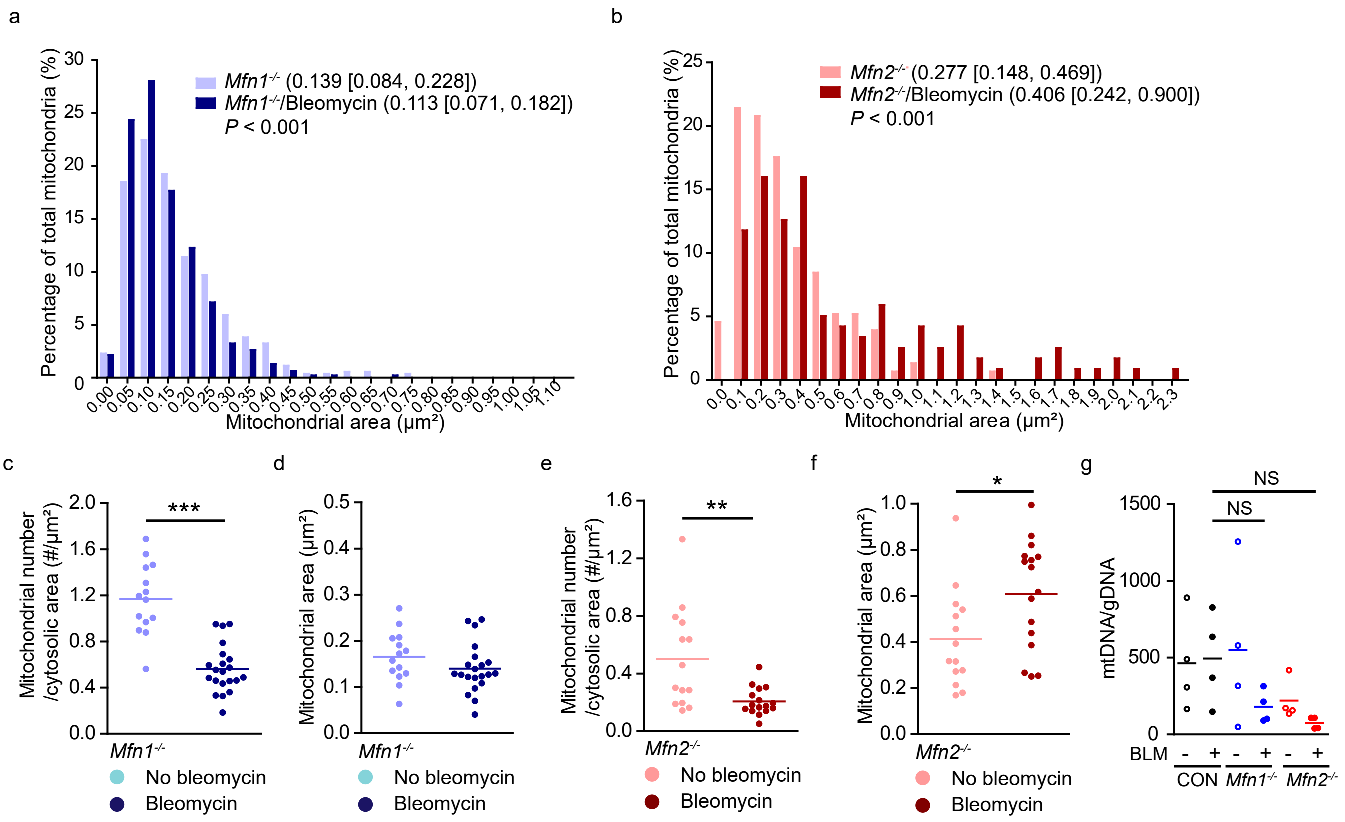

Supplementary Figure 5 Characterization of mitochondrial alterations in AEC2 cells after bleomycin treatment. **a-b** Distribution of the mitochondrial area of each mitochondrion (*Mfn1*$^{-/-}$ mitochondria, no bleomycin n=525, after bleomycin n=464; *Mfn2*$^{-/-}$ mitochondria, no bleomycin n=154, after bleomycin n=119). Data was presented as the median [interquartile range], with comparisons assessed by Mann-Whitney U test). **c-f** Number of mitochondria per µm² of cytosolic area (**c, e**) and the total mitochondrial area (µm²) of each mitochondrion (**d, f**) in each AEC2 cell (**c-f**, each dot represents one AEC2 cell with the line indicating mean; *Mfn1*$^{-/-}$ AEC2 cells, no bleomycin n=14, after bleomycin n=21; *Mfn1*$^{-/-}$ AEC2 cells, no bleomycin n=14, after bleomycin n=16; *p < 0.05, **p < 0.01, ***p < 0.001, vs. no bleomycin by unpaired Student's t-test), using TEM images (12,000X). **g** mtDNA/gDNA copy number ratios as assesses by real-time qPCR in isolated control, *Mfn1*$^{-/-}$, and *Mfn2*$^{-/-}$ AEC2 cells with or without bleomycin treatment (n=4 mice per group; NS, nonsignificant, by one-way ANOVA with post-hoc Bonferroni test). Source data (**a-g**) are provided as a Source Data file.

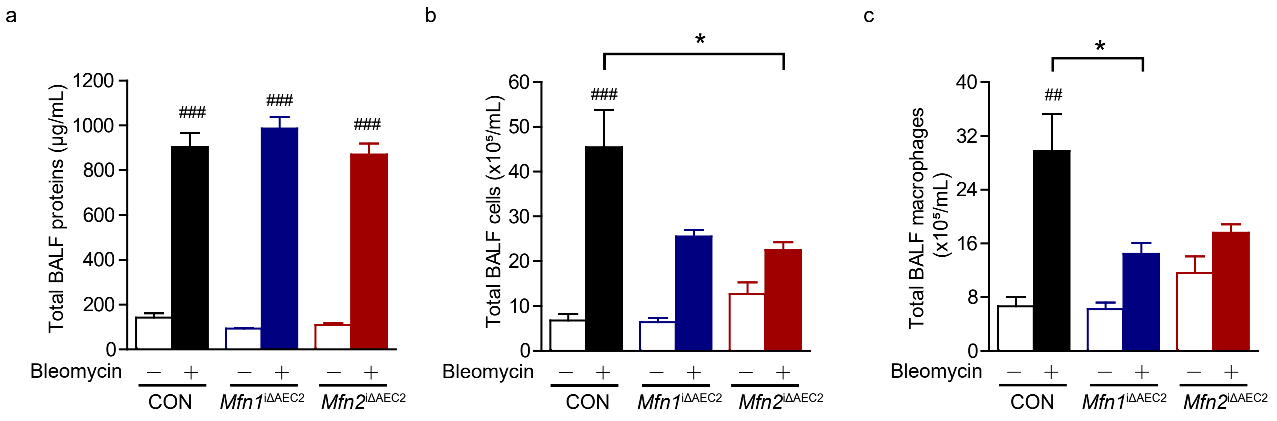

Supplementary Figure 6 Characterization of bronchoalveolar lavage fluid (BALF) obtained from control (PBS n=4, bleomycin n=10), *Mfn1*$^{i\Delta AEC2}$ (PBS n=4, bleomycin n=7) and *Mfn2*$^{i\Delta AEC2}$ (PBS n=4, bleomycin n=10) mice after bleomycin treatment. Total protein (a), total cells (b) and macrophages (c) were measured in the BALF of mice exposed to bleomycin (5 days) or PBS (data presented as mean±s.e.m.; # bleomycin vs. PBS group, * vs. control; *$p < 0.05$, ## $p < 0.01$, ### $p < 0.001$, by one-way ANOVA with post-hoc Bonferroni test). Source data (**a**-**c**) are provided as a Source Data file.

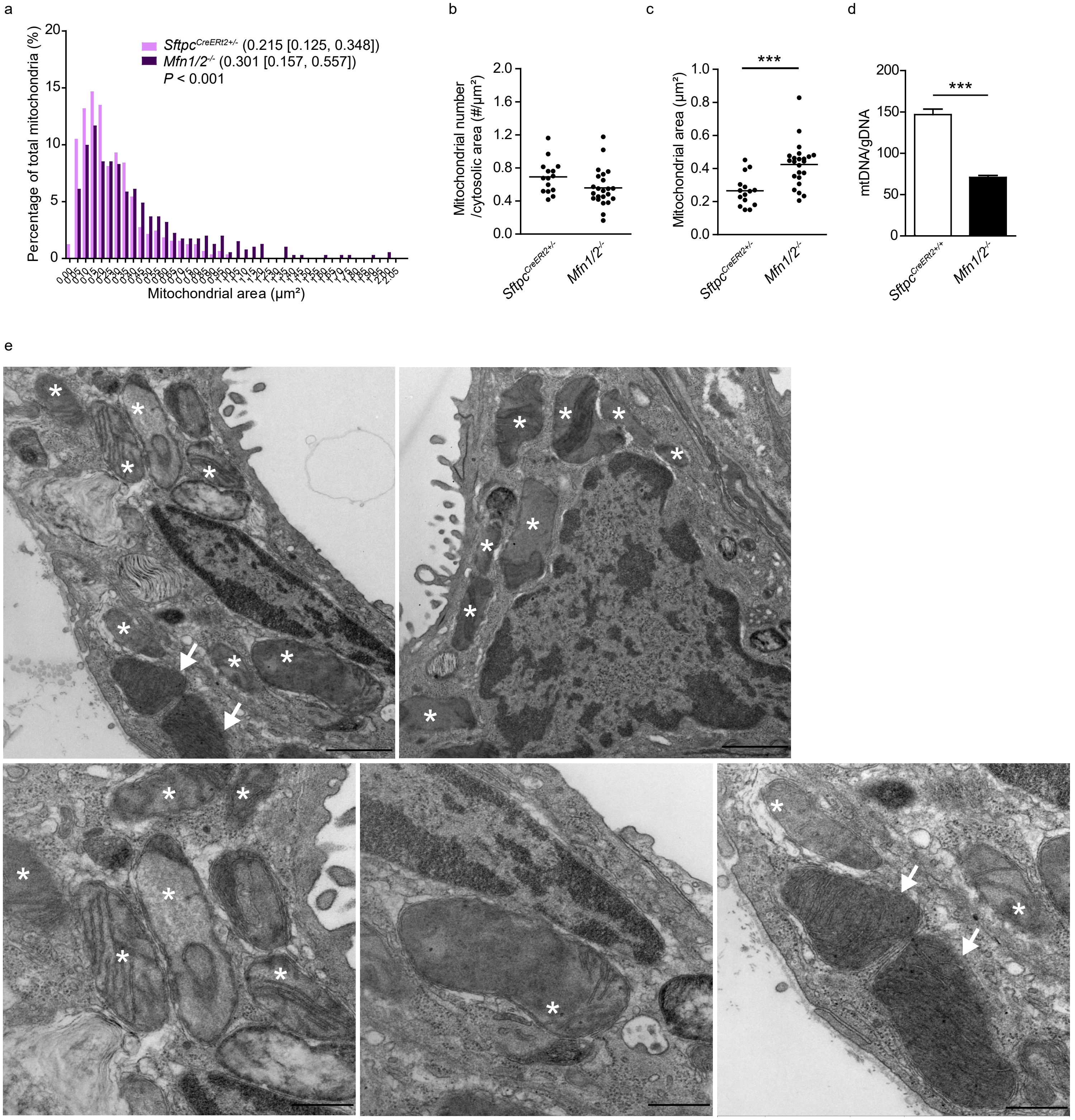

Supplementary Figure 7 Characterization of mitochondrial morphology in *Mfn1/2*$^{-/-}$ AEC2 cells. **a-c** Distribution of the mitochondrial area of each mitochondrion (*Sftpc*$^{CreERT2+/-}$ mitochondria n=335; *Mfn1/2*$^{-/-}$ mitochondria n=413; data presented as the median [interquartile range], and the comparison performed by Mann-Whitney U test) (**a**), and the number of mitochondria per µm2 of cytosolic area (**b**) as well as the individual mitochondrial area (µm²) of each mitochondrion (**c**) in each AEC2 cell (**b-c**, each dot represents one AEC2 cell, with the line indicating mean; *Sftpc*$^{CreERT2+/-}$ AEC2 cells n=15 from 3 mice; *Mfn1/2*$^{-/-}$ AEC2 cells n=23 from 3 mice; ***p < 0.001, vs. *Sftpc*$^{CreERT2+/-}$ AEC2 cells by unpaired Student's t-test), using TEM images (12,000X). **d** Real-time PCR quantification of mtDNA copy number per nuclear genome in *Sftpc*$^{CreERT2+/+}$ and *Mfn1/2*$^{-/-}$ AEC2 cells (n=3 mice per group; data presented as mean±s.e.m.; ***p < 0.001, by unpaired Student's t-test). **e** Representative TEM images (upper panel, 25,000X, scale bar 1 µm; lower panel, 50,000X, scale bar 500 nm) highlight mitochondria with disrupted cristae (marked by asterisk), showing cristae with abnormal morphology or irregular alignment (lower panel, left image), or focal loss of cristae (lower panel, middle image). The arrows mark relatively "normal" mitochondria for comparison (lower panel, right image). Source data (**a-d**) are provided as a Source Data file.

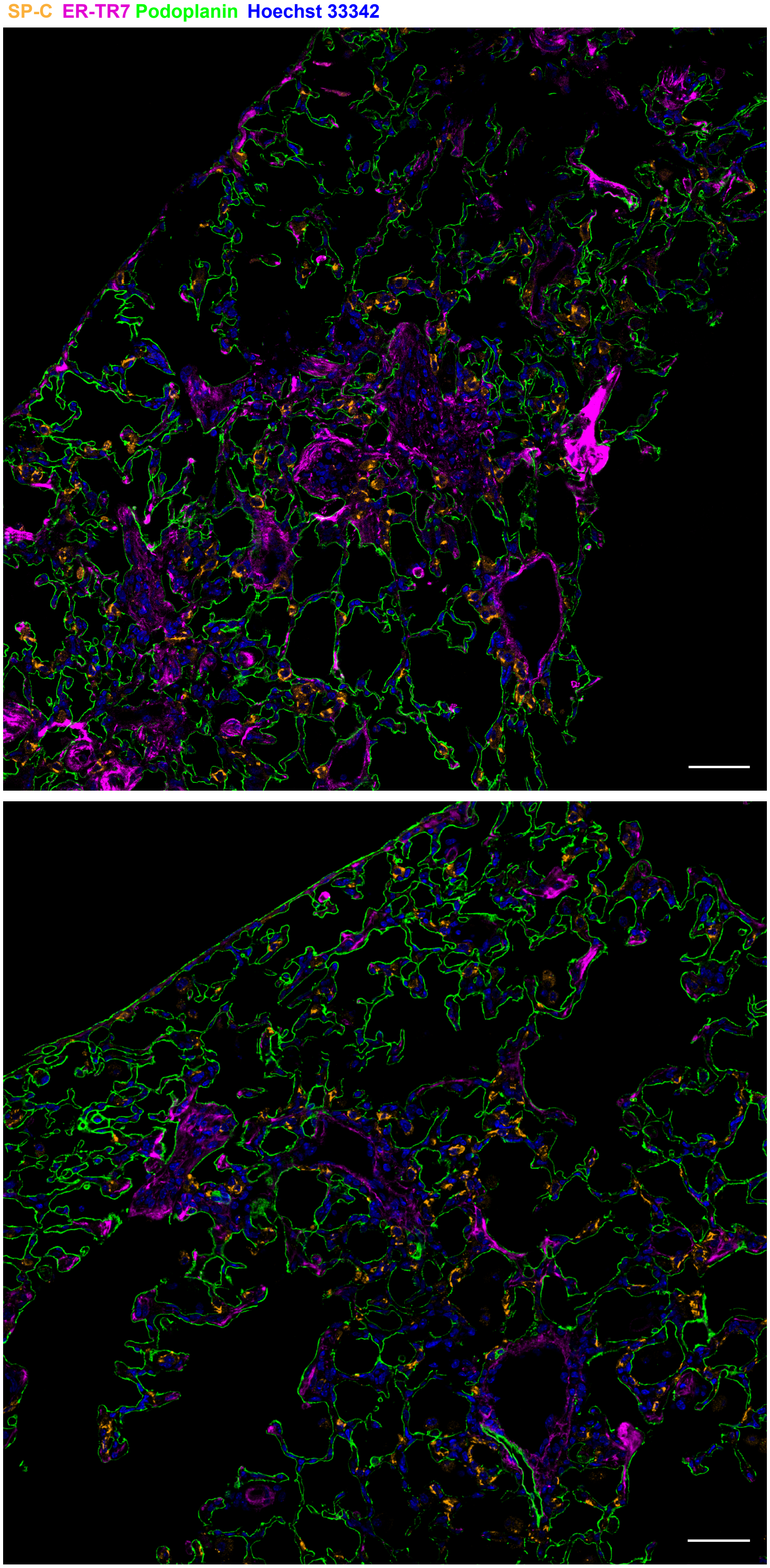

Supplementary Figure 8 Representative immunofluorescent staining, showing 5x5 tiled confocal images (using 40x objective) of frozen *Mfn1/2*$^{i\Delta AEC2}$ mouse lung sections stained for podoplanin (green), surfactant protein-C (SP-C) (yellow), ER-TR7 (magenta), and Hoechst 33342 stain (blue) (scale bar 50 µm).

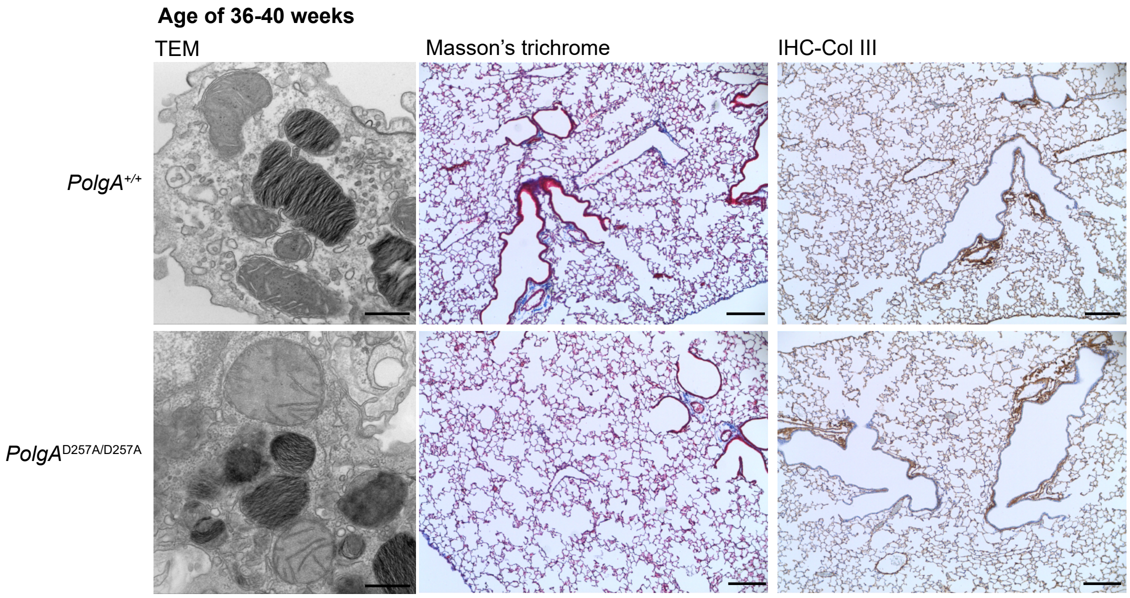

Supplementary Figure 9 Representative TEM images (50,000X) (n=2 mice per groups) and Masson’s trichrome stained lung sections and immunohistochemical staining of collagen III (n=5 mice per group) from control (*PolgA*$^{+/+}$) and *PolgA*$^{D257A/D257A}$ mice (scale bar, left panel, 500 nm; middle and right panel, 200 μm).

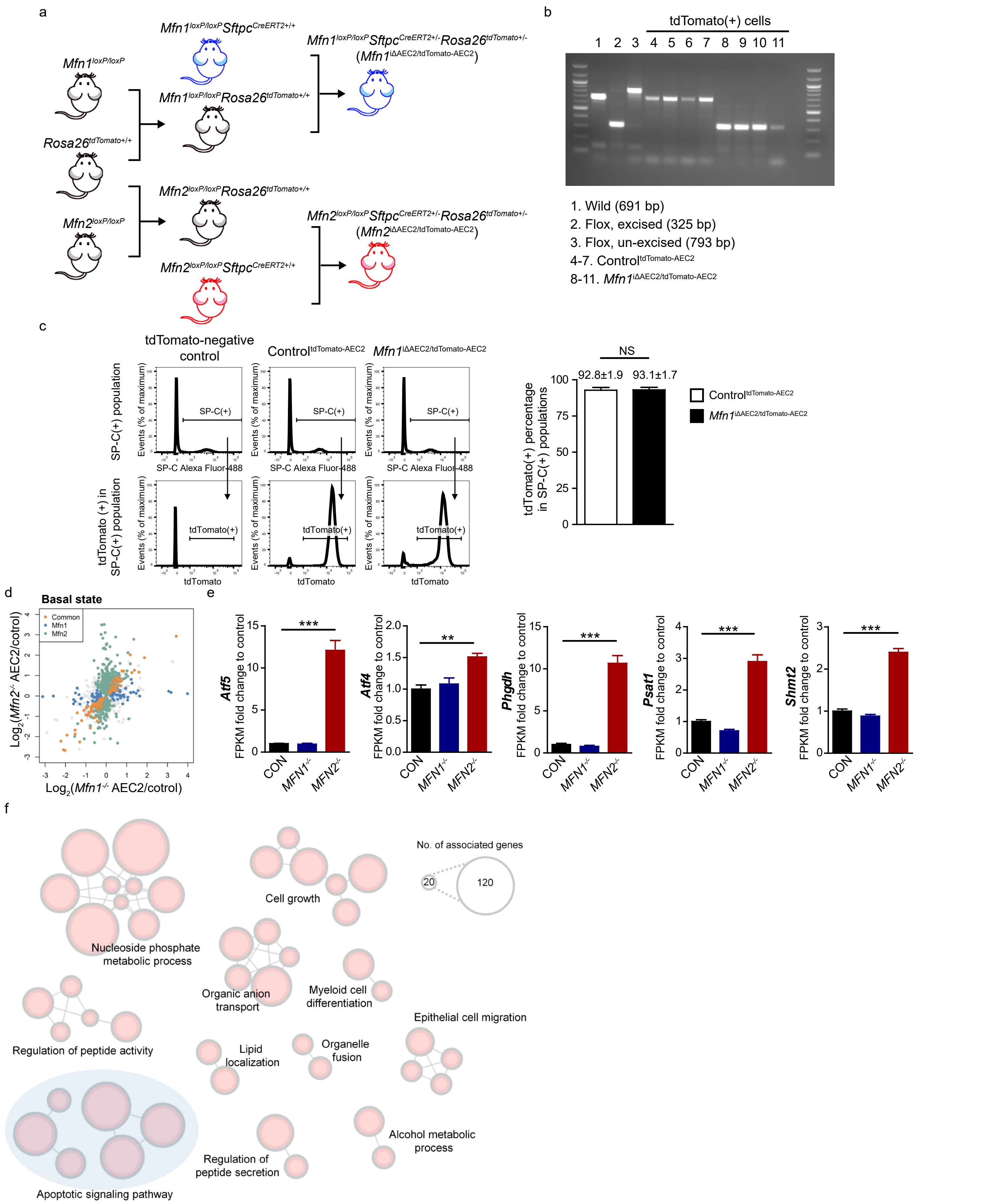

Supplementary Figure 10 Transcriptomic profiles of *Mfn1*- or *Mfn2*-deleted AEC2 cells at baseline **a** Schema demonstrating the generation of *Mfn1*$^{i\Delta AEC2/tdTomato\text{-}AEC2}$ and *Mfn2*$^{i\Delta AEC2/tdTomato\text{-}AEC2}$ mice. **b** Genotyping of tdTomato(+) cells from control$^{tdTomato\text{-}AEC2}$ and *Mfn1*$^{i\Delta AEC2/tdTomato\text{-}AEC2}$ mice 6 weeks after tamoxifen injection (n=4 mice per group; lane 1 to lane 3 serve as positive control). **c** Representative flow cytometry histograms demonstrating the percentage of tdTomato positive SP-C positive AEC2 cells. Data are presented as mean±s.e.m. (**c**, right panel; control$^{tdTomato\text{-}AEC2}$ n=3 mice, *Mfn1*$^{i\Delta AEC2/tdTomato\text{-}AEC2}$ n=4 mice; NS, nonsignificant, by unpaired Student's t-test). **d** Scatterplot to demonstrate genes (orange) that are differentially expressed (adjusted $p < 0.05$) and have the same directional regulation in both *Mfn1*$^{-/-}$ and *Mfn2*$^{-/-}$ AEC2 cells at baseline, when compared to the control. **e** mRNA expression of *Atf4*, *Atf5*, and genes related to de novo serine/glycine synthesis by transcriptome RNA-seq analysis. For each gene, the fold change of FPKM is calculated relative to control. The data are presented as mean±s.e.m. (**adjusted $p < 0.01$, ***adjusted $p < 0.001$ vs. control). **f** Functional enrichment map showing the common GO terms enriched in the differentially expressed genes of *Mfn1*$^{-/-}$ and *Mfn2*$^{-/-}$ AEC2 cells at baseline. Source data (**c**) are provided as a Source Data file.

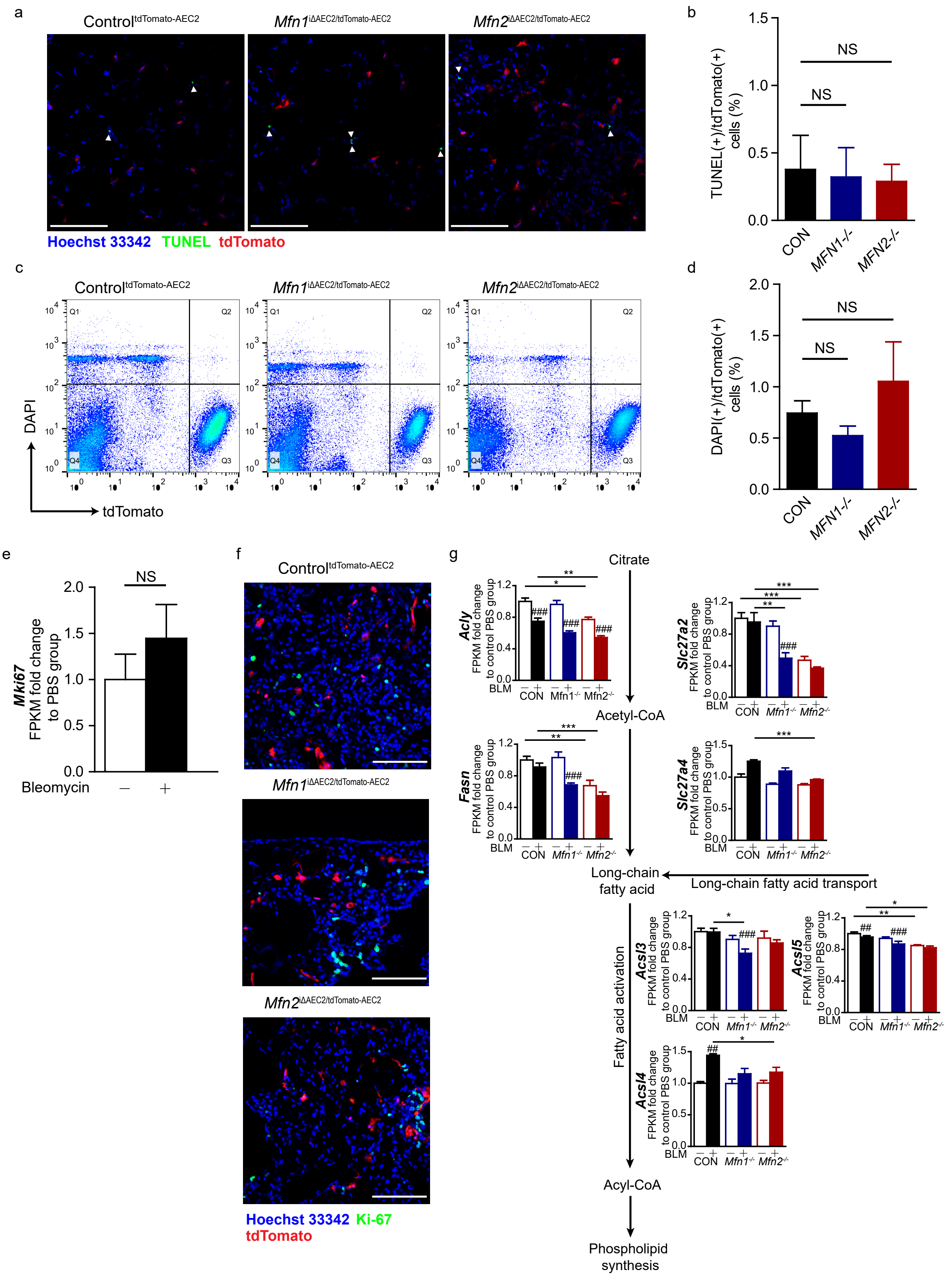

Supplementary Figure 11 Characterizing *Mfn1*- or *Mfn2*-deleted AEC2 cells in bleomycin-induced lung fibrosis model. **a, b** Representative TUNEL staining of murine lung sections obtained 5 days after bleomycin treatment (**a**) with corresponding quantification of TUNEL positive tdTomato(+) cells (**b**). Data are mean±s.e.m. [control (CON) n=10 high-powered fieilds (HPF), *Mfn1*$^{-/-}$ n=12 HPF, *Mfn2*$^{-/-}$ n=10 HPF from 2 mice per group; scale bar 100 μm; NS, nonsignificant, by unpaired Student's t-test). Each HPF was obtained by confocal microscopy using a 25X/0.8 oil immersion objective. **c, d** Representative flow cytometric analysis of whole lung cell suspensions obtained 5 days after bleomycin treatment (**c**) with quantification of the percentage of DAPI-positive cells in the tdTomato(+) population (**d**). Data are mean±s.e.m. (n=4 mice per group; NS, nonsignificant, by unpaired Student's t-test). **e** mRNA expressions of Mki67 in AEC2 cells at day 5 after PBS or bleomycin treatment (n=4 mice per group). The fold change of FPKM was calculated relative to PBS group. The data are presented as mean±s.e.m. (NS, nonsignificant). **f** Representative immunofluorescent staining of Ki-67 (green) and Hoechst 33342 nuclear staining (blue) in lung sections with tdTomato-labeled AEC2 cells, obtained 10 days after bleomycin treatment (n=2 mice per group, and 3 HPF per mice are taken by confocal microscopy using a 25X/0.8 oil immersion objective; scale bar 100 μm). **g** Differential mRNA expression of genes related to fatty acid synthesis, activation and import in pathways identified by functional enrichment analyses in control, *Mfn1*$^{-/-}$ and *Mfn2*$^{-/-}$ AEC2 cells 5 days after PBS or BLM exposure. The fold change of FPKM values were calculated by normalizing to control AEC2 cells with PBS treatment. The data are presented as mean±s.e.m. (# PBS vs. BLM, * knockout vs. control; *adjusted p < 0.05, ## and **adjusted p < 0.01, ### and ***adjusted p < 0.001). Source data (**b**, **d**) are provided as a Source Data file.

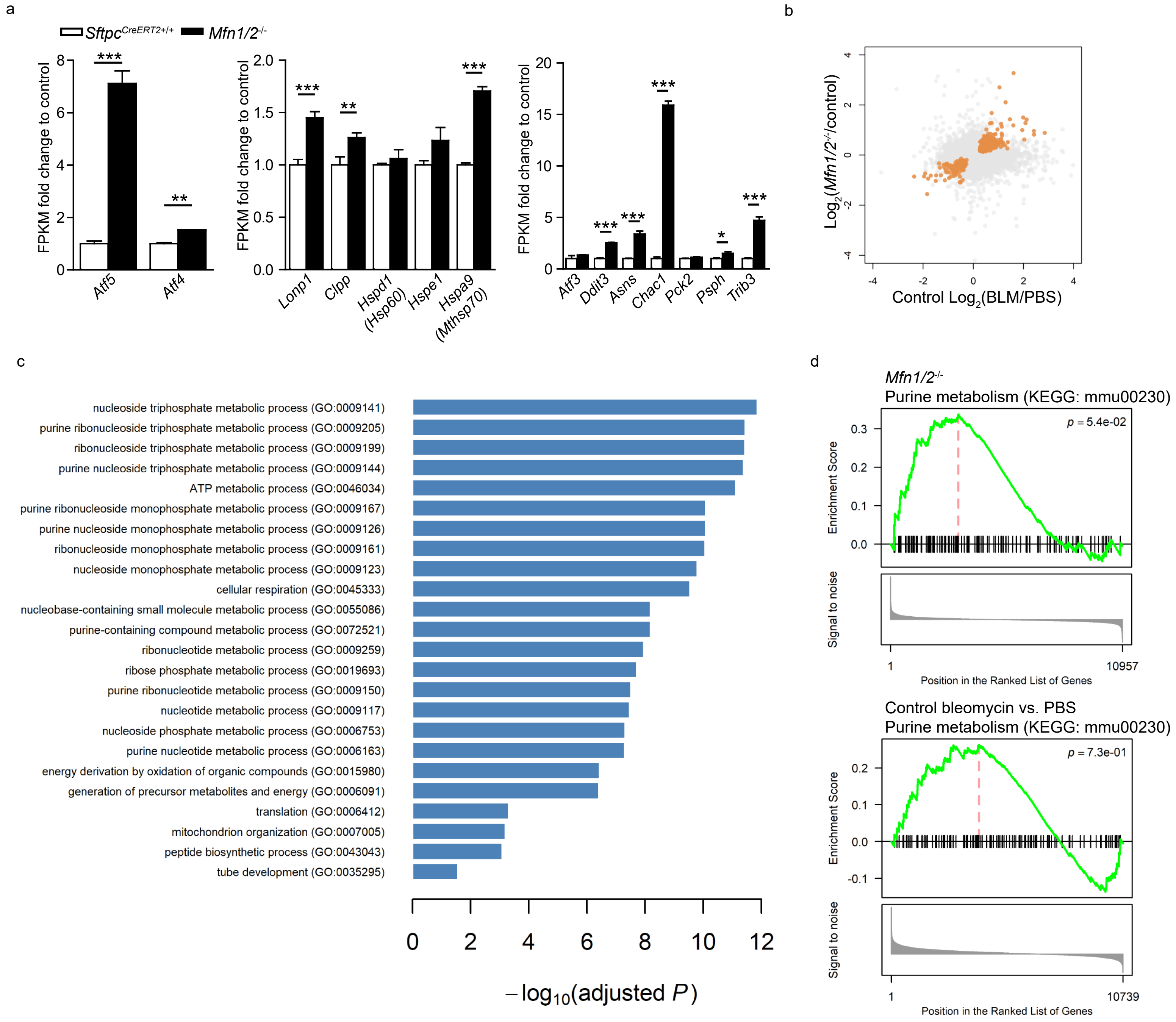

Supplementary Figure 12 AEC2 cells with *Mfn1/2* deletion show transcriptomic upregulation of purine metabolism. **a** mRNA expression of genes related to mitochondrial stress responses. The fold change of FPKM is calculated relative to *Sftpc*$^{CreERT2+/+}$ control. The data are presented as mean±s.e.m. (n=3 mice per group; **adjusted p < 0.01, ***adjusted p < 0.001 vs. *Sftpc*$^{CreERT2+/+}$). **b** Scatterplot showing the common genes (orange) regulated in *Mfn1/2*$^{-/-}$ AEC2 cells at baseline and in AEC2 cells after bleomycin (BLM) treatment, compared to the respective controls. **c** Functional enrichment analyses were then performed on these common genes. **d** Gene-set enrichment analysis (GSEA) based on Kyoto Encyclopedia of Genes and Genomes (KEGG) database revealed upregulated purine metabolism in *Mfn1/2*$^{-/-}$ AEC2 cells at baseline (upper panel) and in AEC2 cells after bleomycin treatment (lower panel).

a

Abbreviation	Lipid name
FC	Free Cholesterol
CE	Cholesterol Ester
AC	Acyl Carnitine
MG	Monoacylglycerol
DG	Diacylglycerol
TG	Triacylglycerol
dhCer	Dihydroceramide
Cer	Ceramide
SM	Sphingomyelin
dhSM	Dihydrosphingomyelin
Sulf	Sulfatide
MHCer	Monohexosylceramide
LacCer	Lactosylceramide
GM3	Monosialodihexosylganglioside
GB3	Globotriaosylceramide
PA	Phosphatidic acid
PC	Phosphatylcholine
PCe	Ether phosphatidylcholine
PE	Phosphatidylethanolamine
PEp	Plasmalogen phosphatidylethanolamine
PS	Phosphatidylserine
PI	Phosphatidylinositol
PG	Phosphatidylglycerol
BMP	Bis(monoacylglycero)phosphate
AcylPG	Acyl Phosphatidylglycerol
LPC	Lysophosphatidylcholine
LPCe	Ether lysophosphatidylcholine
LPE	Lysophosphatidylethanolamine
LPEp	Plasmogen Lysophosphatidylethanolamine
LPI	Lysophosphatidylinositol
LPS	Lysophosphatidylserine
NAPE	N-Acyl Phosphatidylethanolamine
NAPS	N-Acyl Phosphatidylserine
NSer	N-Acyl Serine

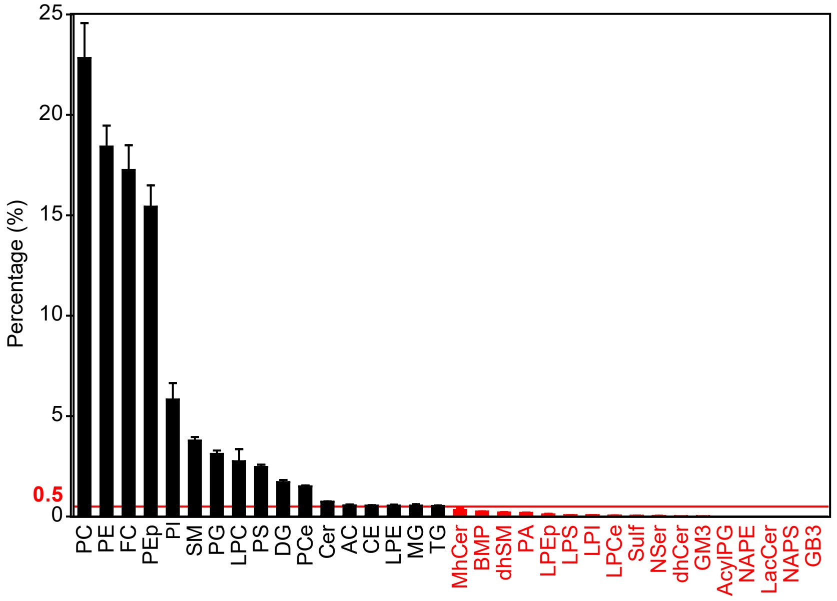

b

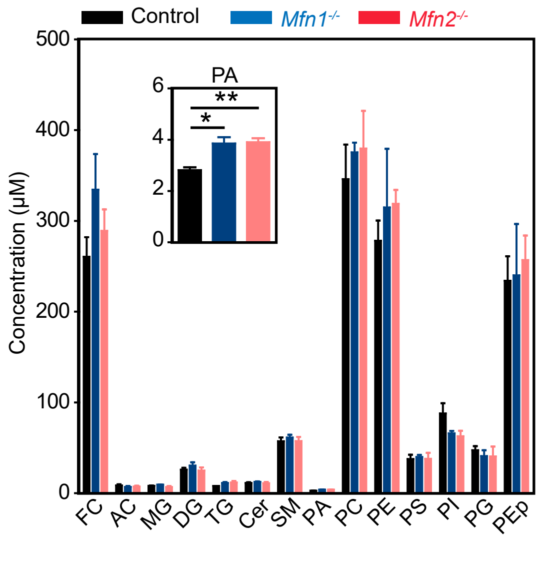

c

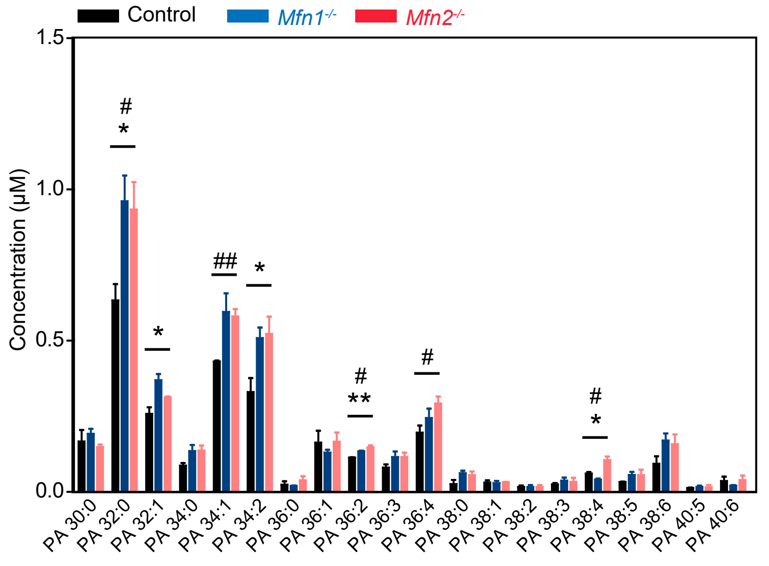

Supplementary Figure 13 Lipidomic in control, *Mfn1*$^{-/-}$ and *Mfn2*$^{-/-}$ AEC2 cells at baseline. **a** The left panel showed the abbreviations of various lipid species, and the right panel showed the percentages of various lipid species in AEC2 cells (n=3 biologically independent samples; lipids with percentage less than 0.5% are marked by red color). **b, c** Lipidomic analysis of lipid species (**b**) and phosphatidic acid species (**c**) present in AEC2 cells from control, *Mfn1*$^{i\Delta AEC2}$ and *Mfn2*$^{i\Delta AEC2}$ mice (n=3 biologically independent samples per group) (* denotes *Mfn1*$^{-/-}$ vs. control, and # denotes *Mfn2*$^{-/-}$ vs. control; * and # $p < 0.05$, ** and ## $p < 0.01$, by unpaired Student's t-test). All the data are presented as mean±s.e.m. (**a-c**). Source data are provided as a Source Data file.

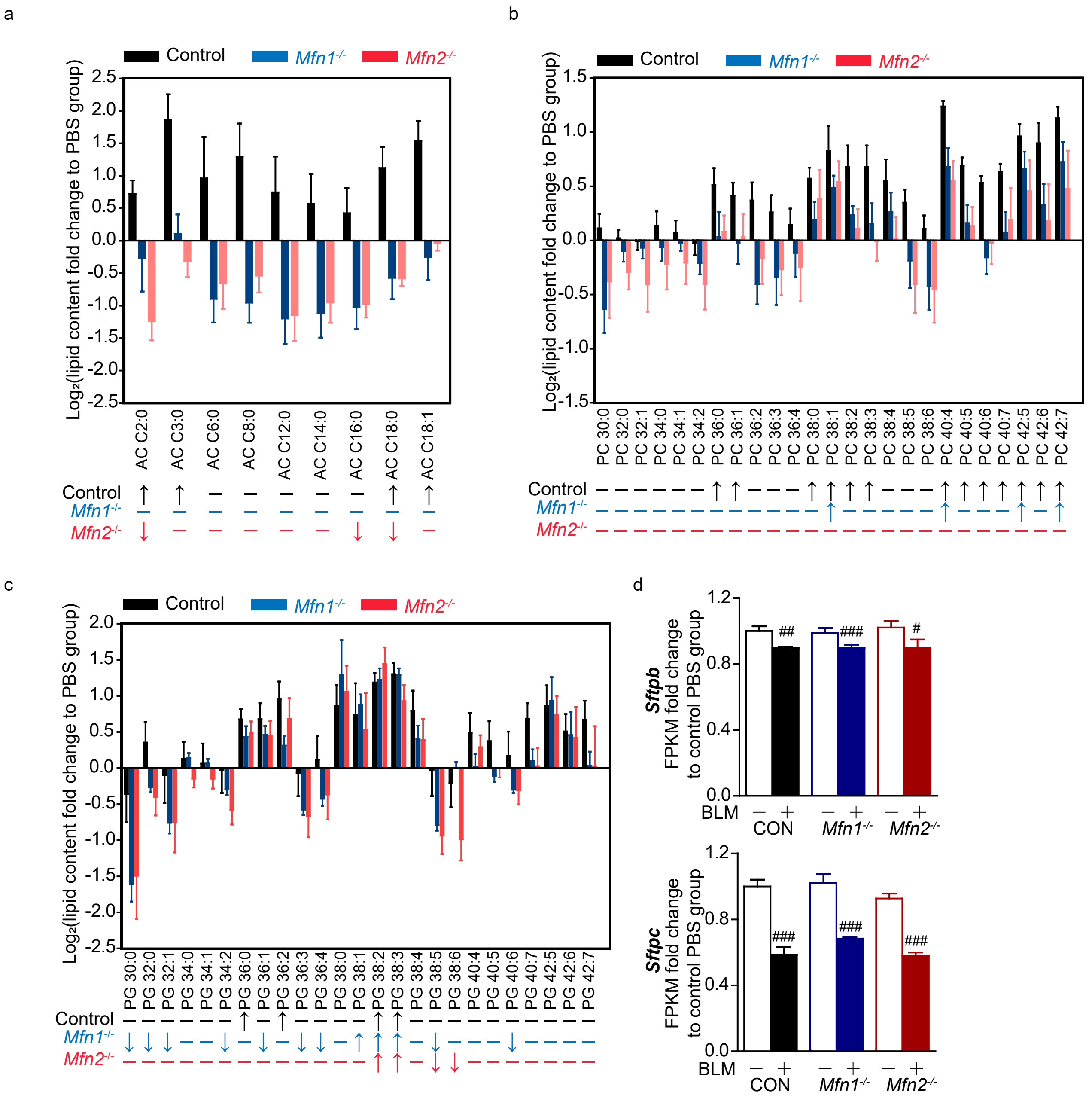

Supplementary Figure 14 Lipidomic and surfactant protein gene expression data in control (CON), *Mfn1*$^{-/-}$ and *Mfn2*$^{-/-}$ AEC2 cells after bleomycin (BLM) treatment. **a-c** Acylcarnitines (**a**), phosphatidylcholine (**b**) and phosphatidylglycerol (**c**) species in control, *Mfn1*- and *Mfn2*-deficient AEC2 cells (n=4 biologically independent samples) 8 days after bleomycin treatment. The fold changes of specific lipid contents in AEC2 cells after bleomycin treatment relative to those after PBS treatment (n=3 biologically independent samples per group) were calculated and log-transformed (base 2) (↑ or ↓, $p < 0.05$, calculated fold changes vs. 1 by unpaired Student's t-test). **d** Surfactant protein genes (*Sftpb* and *Sftpc*) in AEC2 cells at day 5 after PBS or bleomycin treatment (# PBS vs. bleomycin, # adjusted $p < 0.05$, # adjusted $p < 0.01$, and ### adjusted $p < 0.001$). All the data are presented as mean±s.e.m.. Source data (**a-c**) are provided as a Source Data file.

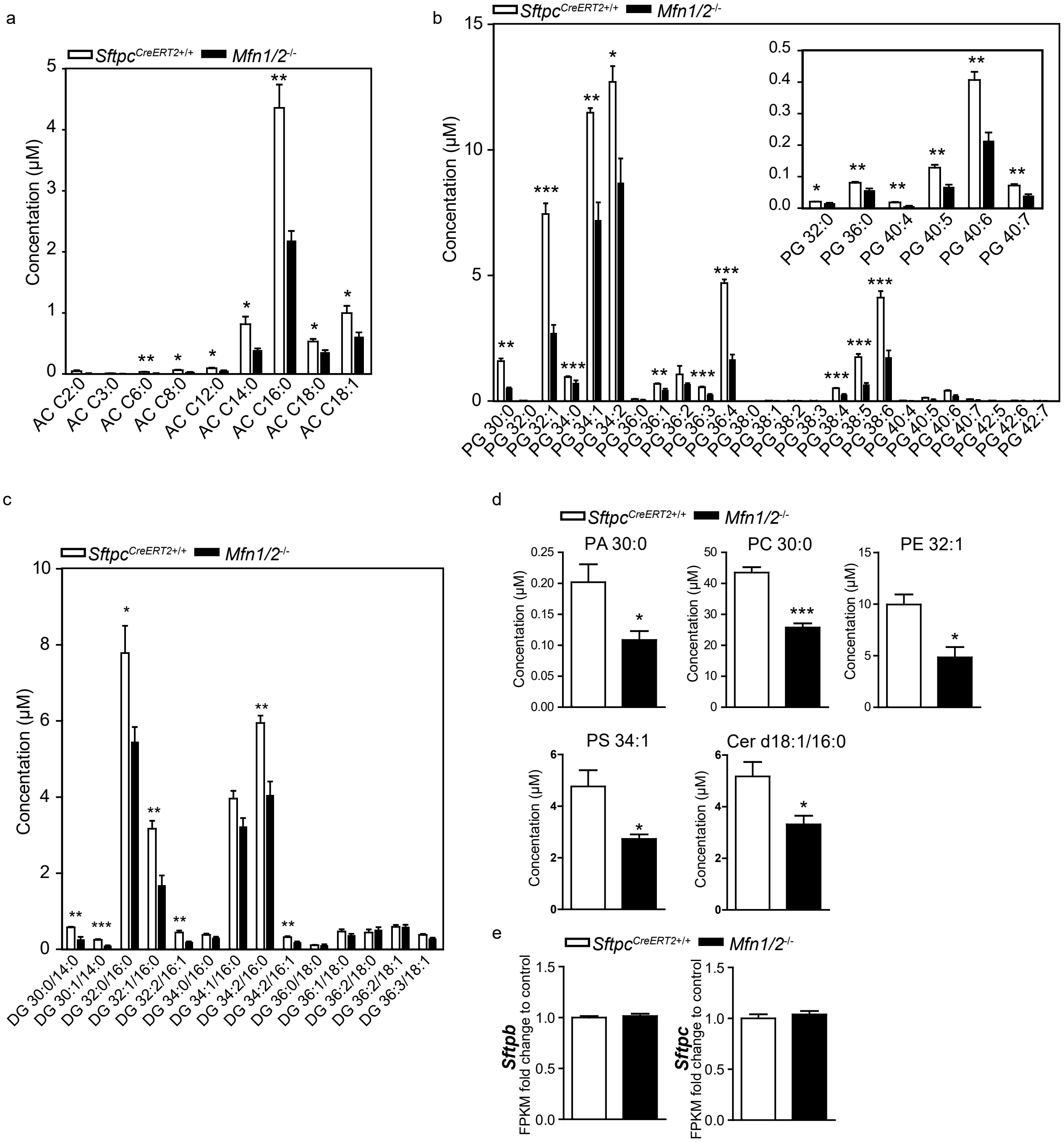

Supplementary Figure 15 Lipidomic profiling and surfactant gene expression in *Mfn1/2*$^{-/-}$ AEC2 cells. **a-d** Lipidomic analysis of acylcarnitine (**a**), phosphatidylglycerol (**b**), diacylglycerol (**c**), and various glycerophospholipids and sphingolipids (**d**) in *Sftpc*$^{CreERT2+/+}$ and *Mfn1/2*$^{-/-}$ AEC2 cells (n=4 mice per group; *p < 0.05, **p < 0.01, ***p < 0.001, vs. *Sftpc*$^{CreERT2+/+}$ by unpaired Student's t-test). **e** mRNA expression of *Sftpb* and *Sftpc* genes. The fold change of FPKM is calculated relative to control (n=3 mice per group). Data are presented as mean±s.e.m. (**a-e**). Source data (**a-c**) are provided as a Source Data file.

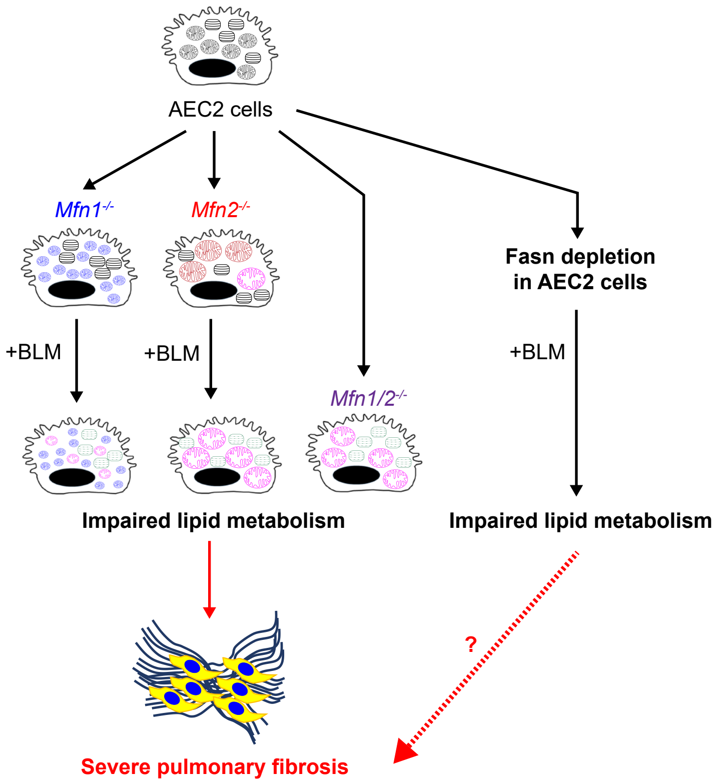

Supplementary Figure 16 Impaired lipid synthesis in AEC2 cells exacerbate lung fibrosis development. Schematic representation showing that loss of MFN1 or MFN2 aggravates lung fibrosis by superimposing abnormal lipid metabolism on extensive mitochondrial damage in AEC2 cells.
