# Supplementary material for: Mitofusins regulate lipid metabolism to mediate the development of lung fibrosis
Source: Nat Commun. 2019 Jul 29;10:3390. doi: 10.1038/s41467-019-11327-1 (PMC6662701; doi:10.1038/s41467-019-11327-1)
Supplement: Supplementary file 2 — Description of Additional Supplementary Files [file 41467_2019_11327_MOESM2_ESM.docx]

**Description of Supplementary Files**

**File Name:** **Supplementary Data 1**

**Description:** Functional enrichment analyses of differential transcripts (adjusted p < 0.001 and fold change > 1.2) between AEC2 cells isolated from mice treated with bleomycin and AEC2 cells from control.

**File Name:** **Supplementary Data 2**

**Description:** Functional enrichment analyses of differential transcripts (adjusted p < 0.05) between Mfn1-/- or Mfn2-/- AEC2 cells and control AEC2 cells at baseline. We further identified the common GO terms enriched in the differentially expressed genes of both Mfn1-/- and Mfn2-/- AEC2 cells.

**File Name:** **Supplementary Data 3**

**Description:** Functional enrichment analyses of differential transcripts (adjusted p < 0.05) between Mfn1-/- or Mfn2-/- AEC2 cells and control AEC2 cells isolated from mouse lungs 5 days after bleomycin treatment. We further identified the common GO terms enriched in the differentially expressed genes of both Mfn1-/- and Mfn2-/- AEC2 cells in the bleomycin-induced lung fibrosis model.

**File Name:** **Supplementary Data 4**

**Description:** Functional enrichment analyses of differential transcripts (adjusted p < 0.05) between Mfn1/2-/- AEC2 cells and control AEC2 cells at baseline.
