# Supplementary material for: Mitofusins regulate lipid metabolism to mediate the development of lung fibrosis
Source: Nat Commun. 2019 Jul 29;10:3390. doi: 10.1038/s41467-019-11327-1 (PMC6662701; doi:10.1038/s41467-019-11327-1)
Supplement: Supplementary file 7 — Reporting Summary [file 41467_2019_11327_MOESM7_ESM.pdf]

Corresponding author(s): Augustine MK Choi
Last updated by author(s): Jun 14, 2019

Reporting Summary

Nature Research wishes to improve the reproducibility of the work that we publish. This form provides structure for consistency and transparency in reporting. For further information on Nature Research policies, see Authors & Referees and the Editorial Policy Checklist.

Statistics

For all statistical analyses, confirm that the following items are present in the figure legend, table legend, main text, or Methods section.

n/a	Confirmed	
☐	☒	The exact sample size (n) for each experimental group/condition, given as a discrete number and unit of measurement
☐	☒	A statement on whether measurements were taken from distinct samples or whether the same sample was measured repeatedly
☐	☒	The statistical test(s) used AND whether they are one- or two-sided<br>*Only common tests should be described solely by name; describe more complex techniques in the Methods section.*
☐	☒	A description of all covariates tested
☐	☒	A description of any assumptions or corrections, such as tests of normality and adjustment for multiple comparisons
☐	☒	A full description of the statistical parameters including central tendency (e.g. means) or other basic estimates (e.g. regression coefficient) AND variation (e.g. standard deviation) or associated estimates of uncertainty (e.g. confidence intervals)
☐	☒	For null hypothesis testing, the test statistic (e.g. F, t, r) with confidence intervals, effect sizes, degrees of freedom and P value noted<br>*Give P values as exact values whenever suitable.*
☒	☐	For Bayesian analysis, information on the choice of priors and Markov chain Monte Carlo settings
☒	☐	For hierarchical and complex designs, identification of the appropriate level for tests and full reporting of outcomes
☒	☐	Estimates of effect sizes (e.g. Cohen's d, Pearson's r), indicating how they were calculated

Our web collection on statistics for biologists contains articles on many of the points above.

Software and code

Policy information about availability of computer code

Data collection	n/a
Data analysis	1. Analyses of TEM Images, immunoblots, and immunofluorescent images were performed using FIJI running Image J software (version 1.52b).<br>2. FlowJo analytical software (version 10) was used for analyses of flow cytometric data.<br>3. For RNA-seq analysis: The raw sequencing reads in binary base call (BCL) format were processed through bcl2fastq 2.19 (Illumina) for FASTQ format conversion and demultiplexing. RNA reads were aligned and mapped to the mm9 mouse reference genome by TopHAEC2 (version 2.0.11), and transcriptome reconstruction was performed by Cufflinks (version 2.1.1). The abundance of transcripts was measured with Cufflinks in Fragments Per Kilobase of exon model per Million mapped reads (FPKM). Differentially expressed genes were identified using the limma package (http://bioconductor.org/packages/release/bioc/html/limma.html). To assess the differential expression, p-values were derived from linear modelling and empirical Bayes moderation and adjusted for multiple testing by the Benjamini-Hochberg method. Gene ontology (GO) over-representation analysis was performed using the clusterProfiler package (http://bioconductor.org/packages/release/bioc/html/clusterProfiler.html). The enrichment maps were visualized by Cytoscape (version 3.6.1), and the functional clusters were highlighted and labeled manually. Heat maps were plotted using Heatmap Illustrator software (Heml 1.0) (hemi.biocuckoo.org) and the pheatmap package (https://github.com/raivokolde/pheatmap), based on the z scores calculated using the gene expressions by FPKM.<br>4. For heatmap generation using lipidomic data, Heatmap Illustrator software (Heml 1.0) (hemi.biocuckoo.org) was used.<br>5. All statistical analyses were performed using SPSS version 17.0 (IBM Corporation) or GraphPad Prism version 5.0 (GraphPad Software)

For manuscripts utilizing custom algorithms or software that are central to the research but not yet described in published literature, software must be made available to editors/reviewers. We strongly encourage code deposition in a community repository (e.g. GitHub). See the Nature Research guidelines for submitting code & software for further information.

Data

Policy information about availability of data

All manuscripts must include a data availability statement. This statement should provide the following information, where applicable:

- Accession codes, unique identifiers, or web links for publicly available datasets
- A list of figures that have associated raw data
- A description of any restrictions on data availability

RNA-seq data have been deposited in Gene Expression Omnibus (GEO) under the accession code GSE115730. All data and methods relevant to the findings of this study are available from the corresponding authors at request. All the TEM and confocal microscopy images in the figures and supplementary figures have been deposited in Mendeley online data repository (doi:10.17632/29v5w97mx4.3). The source data underlying Fig. 1c,d, 2c, e, f, 3c-e, 4a, b, d, 5c, e, 7b-e, 8b-e, and supplementary Fig. 1c-e, 2c, d, 4a-c, f, 5g, 6a-c, 7a-d, 10c and 11b, d are provided as the Source Data file.

Field-specific reporting

Please select the one below that is the best fit for your research. If you are not sure, read the appropriate sections before making your selection.

☒ Life sciences ☐ Behavioural & social sciences ☐ Ecological, evolutionary & environmental sciences

For a reference copy of the document with all sections, see nature.com/documents/nr-reporting-summary-flat.pdf

Life sciences study design

All studies must disclose on these points even when the disclosure is negative.

Sample size	Sample sizes for all experiments were made as large as possible to ensure the robustness of the results. For bleomycin-induced lung fibrosis, many experiments include at least 9 mice, and at least 2-3 individual experiments were performed. Statistics were calculated across all biological and technical replicates. For RNA sequencing analysis, AEC2 cells from at least 3-4 mice per group were isolated. For lipidomic analysis, AEC2 cells from 3-8 mice were isolated to form 3-4 samples. For TEM image analysis, AEC2 cells from 2-3 mice were randomly selected for quantification. All the details about the sample size for each experiment were described in the respective figure legends.
Data exclusions	No data were excluded.
Replication	All the reported findings are based on multiple biological and technical repeats, and can be reproduced without difficulties.
Randomization	For bleomycin-induced lung fibrosis model, mice in different experimental groups were matched for age, gender and weight, and were selected randomly among mice with specific genotypes. Both males and females were used in the study. For image analysis, AEC2 or MLE 12 cells were randomly selected for quantification.
Blinding	The genotyping and experimental conditions, such as bleomycin treatment, of the animals was known to the investigators. The blinding to the experimental conditions is difficult since bleomycin treatment frequently leads to wasting and respiratory distress of the mice, particularly in the knockout mice. For image analysis, such as quantification of the metrics related to mitochondrial morphology, blinding to the condition was attempted as possible to ensure unbiased observations.

Reporting for specific materials, systems and methods

We require information from authors about some types of materials, experimental systems and methods used in many studies. Here, indicate whether each material, system or method listed is relevant to your study. If you are not sure if a list item applies to your research, read the appropriate section before selecting a response.

Materials & experimental systems

n/a	Involved in the study	
☐	☒	Antibodies
☐	☒	Eukaryotic cell lines
☒	☐	Palaeontology
☐	☒	Animals and other organisms
☒	☐	Human research participants
☒	☐	Clinical data

Methods

n/a	Involved in the study	
☒	☐	ChIP-seq
☐	☒	Flow cytometry
☒	☐	MRI-based neuroimaging

Antibodies

Antibodies used	1. For AEC2 isolation through MACS separation: CD45 microbeads (Catalog 130-052-301, Miltenyi Biotec), biotin-conjugated anti-EpCAM antibody (Catalog 13-5791-82, eBioscience), biotin-conjugated anti-EpCAM (1:50; eBioscience), FITC-conjugated anti-biotic antibody (1:10; catalog 130-098-796, Miltenyi Biotec)

2. Immunofluorescent staining of AEC2 cells: SP-C (1: 1000 in blocking buffer, EMD Millipore ABC99), Alexa Fluor-488-conjugated secondary antibody (Thermo Fisher)
3. Immunoblots: mouse MFN1 (1:1000, Antibodies Incorporated 75-162), human MFN1 (1:1000, Proteintech 13798-1-AP), MFN2 (1:1000, Cell Signaling Technology 9482), OPA1 (1:1000, GeneTex GTX48589), DRP1 (1:500, BD Bioscience 611112), FASN (1:1000, Cell Signaling Technology 3180), TIM23 (1:1000, BD Bioscience 611223) and beta-Actin (1:5000, Sigma-Aldrich A2228). The horseradish peroxidase (HRP)-conjugated secondary antibodies, anti-rabbit IgG (1:5000, Santa Cruz sc-2004; GeneTex 213110) and anti-mouse IgG (Santa Cruz sc-2005; BioLegend 405306; both 1:5000).
4. Immunohistochemistry (IHC) staining: vimentin (1:100, Cell Signaling Technology, 5741), alpha-smooth muscle actin (1:640, Cell Signaling Technology, 19245), and collagen III (1:1000, Abcam, ab7778)
5. Immunofluorescent staining: SP-C (1:1000, EMD Millipore ABC99), podoplanin (1:100, R&D Systems AF3244), and Ki-67 (1:500, Abcam ab15580); secondary antibodies against goat IgG (linked to Alexa Fluor-488) or rabbit IgG (linked to Alexa Fluor-488 or Alexa Fluor-568) (1:500, Thermo Fisher), ER-TR7 was conjugated with Alexa Fluor-647 (1:50, Santa Cruz sc-73355 AF647)

Validation

1. For AEC2 isolation through MACS separation: Exp Lung Res 38, 363-373, doi:10.3109/01902148.2012.713077 (2012)
2. For antibodies used for immunoblots, IHC staining, or immunofluoresent staining, the antibodies are validated in the immunoblots reported in this study, in the previous paper (Nature Medicine volume 22, pages 1285–1293 (2016)), or in manufacturers' websites.

Eukaryotic cell lines

Policy information about cell lines

Cell line source(s)	MLE 12 and A549 cells were obtained from ATCC.
Authentication	No specific authentication was used.
Mycoplasma contamination	The cell lines have been tested for mycoplasma infection.
Commonly misidentified lines (See ICLAC register)	No commonly misidentified cell lines were used.

Animals and other organisms

Policy information about studies involving animals; ARRIVE guidelines recommended for reporting animal research

Laboratory animals	Mfn1loxp/loxp (stock 029901-UCD) and Mfn2loxp/loxp (stock 029902-UCD) mice were both generated by David C Chan, and were purchased from Mutant Mouse Resource & Research Centers (MMRRC). SftpcCreERT2+/+ mice were shared from Dr. Brigid Hogan. Fasnloxp/loxp mice were kindly provided by Dr. Clay F Semenkovich, Washington University School of Medicine. ROSA26tdTomato+/+ mice (stock 007914) and PolgA(D257A/D257A) (stock 017341) were purchased from the Jackson Laboratory
Wild animals	No wild animals were used in the experiments.
Field-collected samples	No field-collected samples were used in this study.
Ethics oversight	All animal experiments and procedures were approved by the Institutional Animal Care and Use Committee at Weill Cornell Medicine

Note that full information on the approval of the study protocol must also be provided in the manuscript.

Flow Cytometry

Plots

Confirm that:

☒ The axis labels state the marker and fluorochrome used (e.g. CD4-FITC).

☒ The axis scales are clearly visible. Include numbers along axes only for bottom left plot of group (a 'group' is an analysis of identical markers).

☒ All plots are contour plots with outliers or pseudocolor plots.

☒ A numerical value for number of cells or percentage (with statistics) is provided.

Methodology

Sample preparation	All samples are whole lung single cell suspensions, with or without CD45 negative selection and EpCAM positive selection. In brief, whole lung cell suspension was obtained after dispase digestion, followed by homogenization. DAPI was added for cell viability detection during flow cytometric sorting. Please refer to Online methods for details.
Instrument	Flow cytometric analysis was performed using a LSRFortessa cell analyzer, while flow cytometric cell sorting was performed by an Influx cell sorter (BD Biosciences).

Software	FlowJo analytical software (version 10) was used to analyze flow cytometric data.
Cell population abundance	The cell population abundance depends on the samples for cell cytometric analyses. For CD45(-)EpCAM(+) population, around 94% of the cells are SP-C (+) AEC2 cells. For whole lung cell suspensions, 5%-15% of cells are positive for tdTomato fluorescence, depending on bleomycin treatment or not.
Gating strategy	For all the flow cytometric experiments, FSC and SSC, followed by pulse width gating, were used to set the gate of single cells. For flow cytometric sorting, DAPI staining was used to exclude dead cells, followed by tdTomato gate to capture labeled AEC2 cells. The gating strategies were reported together with each flow cytometric result.

☒ Tick this box to confirm that a figure exemplifying the gating strategy is provided in the Supplementary Information.
